# High Performance Soluble Polyimides from Ladder-Type Fluorinated Dianhydride with Polymorphism

**DOI:** 10.3390/polym10050546

**Published:** 2018-05-18

**Authors:** Fu Li, Jikang Liu, Xiangfu Liu, Yao Wang, Xiang Gao, Xianggao Meng, Guoli Tu

**Affiliations:** 1 Wuhan National Research Center for Optoelectronics, Huazhong University of Science and Technology, Wuhan 430074, China; lifu@hust.edu.cn (F.L.); liujikang@hust.edu.cn (J.L.); xfliu@hust.edu.cn (X.L.); m201772826@hust.edu.cn (Y.W.); 2 School of Materials Science and Engineering, Wuhan Institute of Technology, Wuhan 403052, China; gaoxiang@hust.edu.cn; 3 Key Laboratory of Pesticide and Chemical Biology of the Ministry of Education, College of Chemistry, Central China Normal University, Wuhan 430079, China

**Keywords:** dianhydride, semi-alicyclic, polymorphic, coefficient of thermal expansion, polyimide

## Abstract

A novel rigid semi-alicyclic dianhydride 9,10-difluoro-9,10-bis(trifluoromethyl)-9,10-dihydroanthracene-2,3,6,7-tetracarboxylic acid dianhydride (8FDA) was reported, and its single crystal X-ray diffraction result revealed the existence of the polymorphic structure in this compound. The detail geometric configuration transition during the synthesized process was investigated, exhibiting a transition of from *trans*- to *cis*- when the hydroxyl groups were substituted by fluoride with diethylaminosulfur trifluoride (DAST). Compared with the dianhydride 4,4′-(Hexaflouroisopropylidene) diphthalic anhydride (6FDA) and 1*S*,2*R*,4*S*,5*R*-cyclohexanetetracarboxylic dianhydride (HPMDA), the resulting polyimide (PI) films based on 8FDA exhibited an obviously higher glass transition temperature (*T*_g_, 401 °C) and a much lower coefficient of thermal expansion (CTE, 14 ppm K^−1^). This indicates that 8FDA is an ideal building block in high-performance soluble PIs with low CTE.

## 1. Introduction

Recently, the demand of high-performance transparent polymer substrates increases dramatically with the rapid development of flexible electronics, especially for the flexible OLED displays [1,2,3]. Because of the severe conditions during the TFT fabrication for OLED displays, the ideal flexible transparent substrates require a high *T*_g_ over 370 °C, a small CTE value lower than 20 ppm K^−1^, high transparency, and so on [4]. Due to their outstanding chemical and irradiation resistance, high thermal stability, and excellent optical and mechanical properties, transparent PI films became the best choice for transparent substrates in flexible OLED displays [5,6,7,8,9].

The traditional aromatic polyimides (ArPIs) are usually with the color of yellow or brown [10,11,12,13,14]. The color originated from the electron transfer between the donor and the acceptor in the backbone of ArPIs, usually induced by the inter- and intra-chain charge transfer complex (CTC) effect, the strong conjugation, and so on. Besides, the ArPIs also exhibit poor processability and solubility [15,16,17,18,19,20], which limits their application in the flexible optoelectronic field [6,7,21,22,23,24,25]. In order to obtain suitable PI substrates, researchers have to overcome these disadvantages.

6FDA is a well-known building block for transparent PIs. The sp^3^ hybrid quaternary carbon in 6FDA increases the degree of molecular distortions and reduces the conjugation length in the backbone. The steric congestion caused by the CF_3_ group disrupts the chain packing and decreases the intermolecular interaction [11,19,26]. Then, the formation of charge-transfer complex (CTC), which will result in a deep yellow color in the PI films, is suppressed, and then the transparency of the PI films is improved [13]. However, the flexibility of 6FDA will lead to a large linear thermal expansion coefficient (CTE) value of about 50 ppm K^−1^ and a lower *T*_g_ of less than 350 °C [27]. The introduction of alicyclic segments in the backbone of PIs was another effective method of the synthesis transparent PIs. The 1,2,4,5-cyclohexanetetracarboxylic dianhydride (HPMDA) is widely used to prepare transparent PIs [28,29]. However, the HPMDA-based PI films suffer from high CTE over 50 ppm K^−1^ and weak antioxidant capacity, and are prone to yellow stain under high temperatures [28,30,31]. So, it is necessary to develop new dianhydride monomers to satisfy the urgent need of flexible OLED displays.

Considering the integrated advantages of 6FDA, and HPMDA that are mentioned above, the low dimension stabilities of PI are derived from alicyclic structure-modified dianhydrides and diamines [28,29,30,32,33,34]. Herein, a rigid semi-alicyclic dianhydride 8FDA was developed to prepare high-performance, transparent PIs. The rigid structure of 8FDA was expected to increase the *T*_g_ and decrease the CTE at the same time. Additionally, the 1,4-cyclohexadiene segments in 8FDA can break the conjugation of the polymer chain and increase the free volume, which will decrease the charge transfer absorptions and improve the optical properties. The ladder structure of 8FDA will improve the solubility of 8FDA-based PIs. The possible reaction mechanism of 8FDA and the geometric configuration transition processes during the synthesis was investigated in detail. Additionally, the single crystal X-ray diffraction test proved that 8FDA is a novel polymorphic crystal [35].

## 2. Experimental

### 2.1. Materials

Dianhydrides, 4,4′-(hexaflouroisopropylidene) diphthalic anhydride (6FDA), 1*S*,2*R*,4*S*,5*R*-cyclohexanetetracarboxylic dianhydride (HPMDA). Diamines, 2,2′-bis(trifluoromethyl)-[1,1′-biphenyl]-4,4′-diamine (TFDB), and 4,4′-oxybis(3-(trifluoromethyl)aniline) (TFODA) were purchased from Merck and sublimated before use. Commercially available *N*-methyl-2-pyrrolidinone (NMP), m-cresol, and *N*,*N*-dimethylacetamide (DMAc) were purified by vacuum distillation over P_2_O_5_, and *N*,*N*-dimethylformamide (DMF) was purified by vacuum distillation over CaH_2_ and then stored over 4 Å molecular sieves prior to use. Other commercially available materials were used without further purification.

### 2.2. Instrumentation

IR spectra were recorded on a Perkin Elmer Fourier Transform Infrared spectrometer Perkin Elmer SP one FT-IR (PerkinElmer, Waltham, MA, USA). ^1^H NMR, ^13^C NMR, and ^19^F NMR spectra were measured on a Bruker DRX 600 spectrometer with CDCl_3_ and DMSO-*d*_6_ as the solvent and tetramethylsilane as the internal reference. Single crystal diffraction was measured on BRUKER, SMART APEX CCD. The melting points were measured with a microscopic melting point meter SGW-X4. Elemental analyses were carried out on a Perkin-Elmer-240 (11) C, H, N analyzer. Wide-angle X-ray diffraction (WAXD) measurement were performed at 25 °C on a Bede XRD Di system, using graphite-monochromatized Cu-Kα radiation (λ = 0.15405 nm). Ultraviolet visible (UV-vis) spectra of the polymer films were recorded on a Shimadzu UV-visible spectrophotometer UV-2450 (Shimadzu, Kyoto, Japan). Thermogravimetric analysis (TGA) was conducted with Perkin-Elmer TGA-2 in flowing nitrogen at a heating rate of 10 °C min^−1^. Dynamic mechanical analysis was conducted with DMA Q800 V20.22 Build 41 using tensile mode at the frequency of 1 Hz. Thermal mechanical analysis (TMA) was characterized through TMA Q400. An Instron universal tester model 1122 (GB/T1040.1-2006) was used to study the stress-strain behavior of the PIs film samples at room temperature with a stretching rate of 2.5 mm min^−1^, and the data was the average value of five experiments excluding the maximum and the minimum values. Contact angle measurements were carried out using Drop Master 300 Contact AM system (Kyowa Interface Science Co., Saitama, Japan). Atomic force microscope (AFM) images were measured by Veeco Dimension 3100 NanoScope in a tapping mode with Bruker RTESPA-300 probes (Bruker Nano Inc., Karlsruhe, Germany). The refractive indices of the pure polyimide films were measured with a dual rotating-compensator Mueller matrix ellipsometer 38-40 (ME-Lellipsometer, Wuhan Eoptics Technology Co., Ltd., Wuhan, China), and the Cauchy model was used in the tests.

### 2.3. Monomer Synthesis of 8FDA

**Scheme 1:**
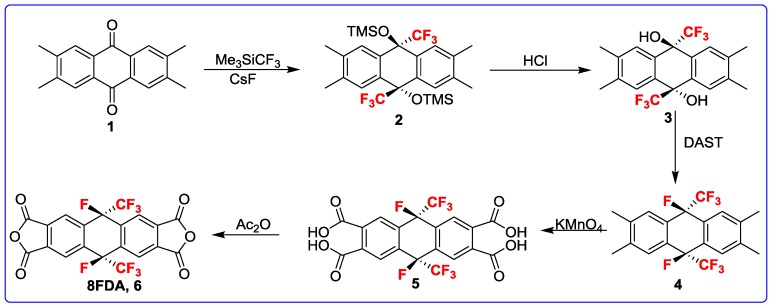
Synthetic route to 8FDA.

8FDA was prepared by five-step synthesis, as shown in Scheme 1.

*((2,3,6,7-Tetramethyl-9,10-bis(trifluoromethyl)-9,10-dihydroanthracene-9,10-diyl) bis(oxy))bis(trimethylsilane)* (Compound **2**): Compound **1** (2,3,6,7-tetramethylanthracene-9,10-dione (30.00 g, 113.5 mmol, 1.000 equiv.)) was suspended in tetrahydrofuran (THF 500.0 mL) at r.t. and trimethyl(trifluoromethyl)silane (42.80 mL, 283.8 mmol, 2.500 equiv.) was added. The suspension was cooled to 0 °C and CsF (172.4 mg, 0.1300 mmol, and 0.01000 equiv.) was added. After stirring for 10 min, the reaction mixture was warmed up to r.t., and stirring was continued for 24 h. The reaction mixture was filtered, and the remaining yellow solid was rinsed with *n*-hexane (50.00 mL). The crude product was purified by column chromatography on silica (*n*-pentane). Compound **2** was obtained as white solid (18.68 g, 34.00 mmol, 30%). M.p.: 230–232 °C; ^1^H NMR (600 MHz, CDCl_3_, ppm) δ 7.67 (s, 4H), 2.38 (d, *J* = 3.3 Hz, 12H), −0.12 (d, *J* = 14.4 Hz, 18H); ^13^C NMR (101 MHz, CDCl_3_, ppm) 137.56, 137.22, 131.04, 130.69, 130.40, 125.87, 19.67, 2.19, 1.99, 1.54. ^19^F NMR (565 MH, CDCl_3_, ppm) δ −78.39 (s, 6F).

*(2,3,6,7-Tetramethyl-9,10-bis (trifluoromethyl)-9,10-dihydroanthracene-9,10-diol)* (Compound **3**): Compound **2** (20.00 g, 36.40 mmol, 1.000 equiv.) was dissolved in 80.00 mL THF and heated to reflux temperature. Concentrated hydrochloric acid was slowly added, and the reaction remained in reflux for 3 h. After being cooled to room temperature, the mixture was suction filtered. The filter cake was washed three times with water and three times with n-hexane. The product was dried in vacuum at 100 °C. Compound **3** was obtained as colorless crystal with a yield of 95%. m.p.: 287–290 °C. ^1^H NMR (600 MHz, DMSO-*d*_6_, ppm) δ 7.69 (d, *J* = 1.2 Hz, 4H), 7.32 (s, 2H), 2.32 (s, 12H). ^13^C NMR (400 MHz, DMSO-*d*_6_, ppm) δ 137.26 (s), 131.90 (s), 129.46 (s), 72.11 (t, *J* = 26.6 Hz), 19.84 (s). ^19^F NMR (565 MHz, DMSO-*d*_6_, ppm) δ −76.69 (s, 6F).

*9,10-Difluoro-2,3,6,7-tetramethyl-9,10-bis(trifluoromethyl)-9,10-dihydroanthracene* (Compound **4**): Compound **3** (10.00 g, 24.70 mmol, 1.000 equiv.) was cooled to −78 °C under anhydrous anaerobic conditions in 30.00 mL of anhydrous THF, and diethylaminosulfur trifluoride (DAST) (7.230 mL, 54.40 mmol, 2.200 equiv.) was added drop wise. After it was completely reacted, sodium bicarbonate was added to quench the excess DAST. The crude product was purified by silica column chromatography on silica gel with n-hexane as the eluent, resulting in colorless solid (Compound **4**, 85% yield). m.p.: 203–206 °C). ^1^H NMR (600 MHz, CDCl_3_, ppm) δ 7.68 (d, *J* = 26.0 Hz, 4H), 2.39 (s, 12H). ^13^C NMR (101 MHz, CDCl_3_, ppm) δ 139.87, 139.61, 129.18, 128.90, 127.44, 126.52, 19.90. ^19^F NMR (565 MHz, CDCl_3_, ppm) δ −77.80 (d, 2F), −77.98 (m, 4F), −157.55 (m, 2F).

*9,10-Difluoro-9,10-bis(trifluoromethyl)-9,10-dihydroanthracene-2,3,6,7-tetra-carboxylic acid* (Compound **5**): Compound **4** (5.000 g, 12.20 mmol, 1.000 equiv.) was dissolved in 30.00 mL of mixture of pyridine and water with the volume ratio of 1:1, then the mixture was heated to reflux. 19.34 g, (0.1220 mmol, 10.00 equiv.) potassium permanganate was added into the reaction flask in portions. After it was completely reacted, the mixture was filtered through hot water, and the filtrate was concentrated. The product was dissolved in hot water, and concentrated hydrochloric acid was added to get the crude product. The crude product was recrystallized by acetic acid. Pure product was obtained with a yield of 85%, m.p.: 289–291 °C. ^1^H NMR (600 MHz, DMSO-*d*_6_, ppm) δ 13.98 (s, 4H), 8.53–7.94 (m, 4H). ^13^C NMR (101 MHz, DMSO-*d*_6_, ppm) δ 167.05, 166.97, 136.90, 136.79–136.53, 130.00, 129.40. ^19^F NMR (565 MHz, DMSO-*d*_6_, ppm) δ −76.31 (m, 6F), −142.23 (m, 2F).

*9,10-Difluoro-9,10-bis(trifluoromethyl)-9,10-dihydroanthracene-2,3,6,7-tetracarboxylic acid dianhydride* (8FDA): Compound **5** (5.000 g, 9.470 mmol) was dissolved in 30.00 mL acetic anhydride, then heated to reflux. After the reaction completed, the solution was concentrated. The crude product was recrystallized in the mixture of 30.00 mL toluene and 3.000 mL acetic anhydride. Pure 8FDA was obtained (85%), m.p.: 295–298 °C. Elem. Anal. Calcd. for C_20_H_4_F_8_O_6_ (%): C, 48.80; H, 0.82; F, 30.88; O, 19.50. Found: C, 48.92; H, 0.84. F, 30.87; O, 19.37. ^1^H NMR (600 MHz, DMSO-*d*_6_, ppm) δ 8.83–8.63 (m, 1H), 8.34 (dd, *J* = 43.5, 25.8 Hz, 1H). ^13^C NMR (101 MHz, DMSO-*d*_6_, ppm) δ 161.87, 161.80, 135.42, 134.13, 125.73, 125.64. ^19^F NMR (565 MHz, DMSO-*d*_6_, ppm) δ −75.64 (m, 6F), −139.35 (s, 2F).

The ^1^H, ^13^C, ^19^FNMR, and FT-IR spectra of monomers were shown in Figures S1–S16). Crystal data was summarized in Table S1.

### 2.4. Polymer Preparation

**Scheme 2:**
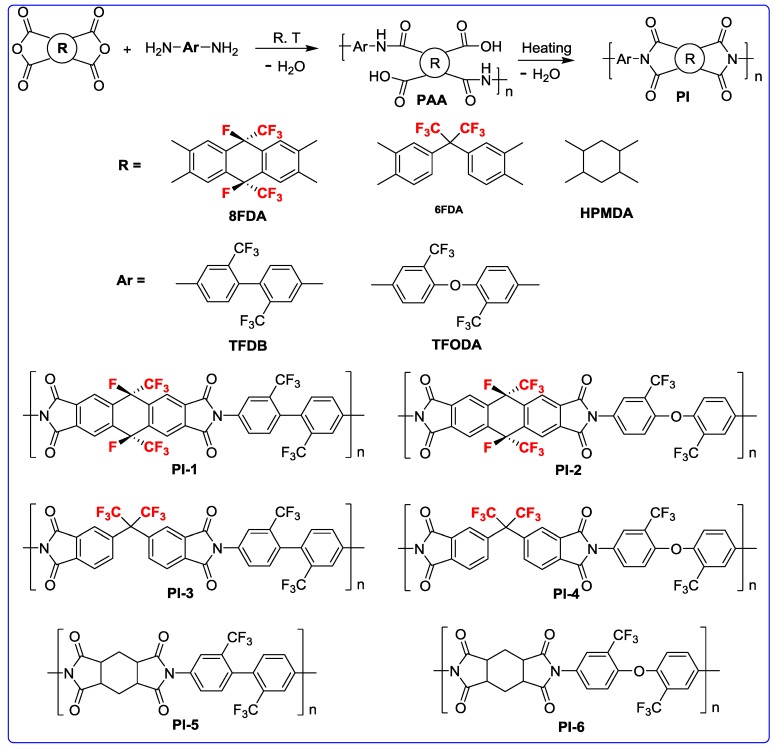
Synthesis of polyimides based on 8FDA, 6FDA, and HPMDA.

**Figure 1:**
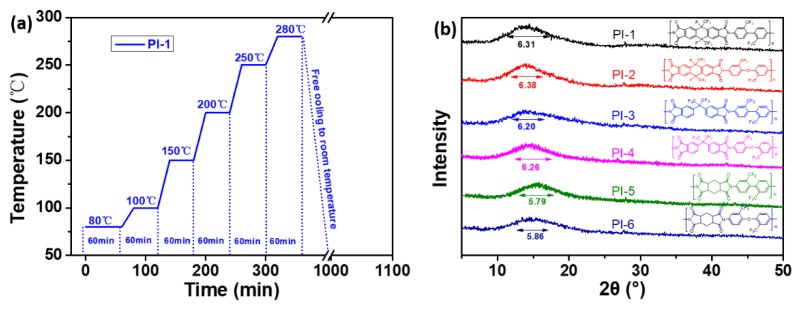
(**a**) The preparation process of PI films and (**b**) WAXD patterns of polyimides films.

A series of polyimide films was prepared by conventional thermal imidization of 8FDA, 6FDA, and HPMDA with TFDB and TFODA, respectively. For convenience, the PI films derived from 8FDA, 6FDA, and HPMDA are coded as PI-x. The synthesis of the polyimides based on 8FDA, 6FDA, and HPMDA were shown in Scheme 2. The synthesis of PI-4 (6FDA/TFODA) was used as an example to illustrate the general thermal imidized synthetic procedure to prepare the polyimide films. TFODA (1.010 g, 3.004 mmol) was dissolved in 13.11 g dried DMAC in a 50.00 mL flask, and then 6FDA (1.321 g, 2.974 mmol) was added to the solution in one portion. The mixture was stirred at room temperature for 24 h to obtain a viscous poly(amic acid) (PAA) solution. Then, the PAA solution was poured onto a clean glass plate, followed by heating at 80 °C for 1 h for the slow release of the solvent, and sequential heating at 100 °C for 1 h, 150 °C for 1 h, 200 °C for 1 h, 250 °C for 1 h, and 280 °C for 1 h. The PAA was subsequently converted into PI-4 film by thermal imidization process. Then, it was peeled from the glass surface after being soaked in deionized water. IR (cm^−1^, film): 1781, 1728 (imide carbonyl asym. and sym. stretching), 1385 (C–N stretching), 1121, and 727 (imide ring deformation). The processing procedure is shown in Figure 1a; FT-IR spectra and photos of PI films are shown in Figure S17.

## 3. Results and Discussion

### 3.1. Monomer Synthesis

8FDA was prepared by a five-step synthesis, as shown in Scheme 1. The isolated yield for the first step reaction was about 30%, which needs to be further improved. For the other steps 2–5, all the isolated yields were higher than 80%. The structure of 8FDA was confirmed via FT-IR; elemental analysis; single-crystal X-ray diffraction; and ^1^H, ^13^C, and ^19^F NMR, as shown in Figures S1–S16. The crystal data was summarized in Table S1. The possible mechanism is depicted in the supporting information and shown in Scheme S1.

### 3.2. Description of Crystal Structure of Compounds ***2***–***4***, and 8FDA (***6a***, ***6b***, and ***6c***)

The crystal data of all the six compounds are summarized in Table S1. They were crystallized in the triclinic (2), monoclinic (**4**, **6a**, **6b,** and **6c**) and tetragonal space groups, respectively. In Compounds **2** and **3**, their asymmetric units both consisted of one half of their molecules (Figure S18a,b). Additionally, each complete molecule exists in the asymmetric units of compounds **4**, **6b,** and **6c** (Figure S18c,e,f). For **6a**, there are two independent 8FDA molecules in its asymmetric unit (Figure S18d). For **6c**, two toluene molecules were incorporated into the crystal lattice (Figure S18f)). As the three polymorphs of 8FDA, the dihedral angles between two lateral benzene rings in one molecule (21.1(1)o/24.0(1)o) in **6a** are apparently smaller than those in **6b** (27.9(1)o) and **6c** (26.8(1)o). Thus, the reactivity of 8FDA may be mediated by its various morphologies in the synthesis of PI-1 and PI-2. Except for this, the bond and angle parameters in the parent structures of these six crystals are common to some analogs [35].

As for their crystal packing in compounds **2**–**4**, some weak molecular interactions such as O–H…O, C–H…π, C–H…F, and F…F have been observed (Figures S19–S21). According to the raw materials, the two trifluoromethyl groups in compounds **2**–**3** are both expectedly residing at the trans conformations. However, in the subsequent compounds **4** and 8FDA (**6a**–**c**), they are all surprisingly re-constructed into a cis arrangement via a proposed mechanism (Scheme S1 and vide infra). What is more, the intra-molecular conformation flexibility and inter-molecular interaction characteristics of 8FDA may also illustrate the reaction mechanism to a certain extent.

**Figure 2:**
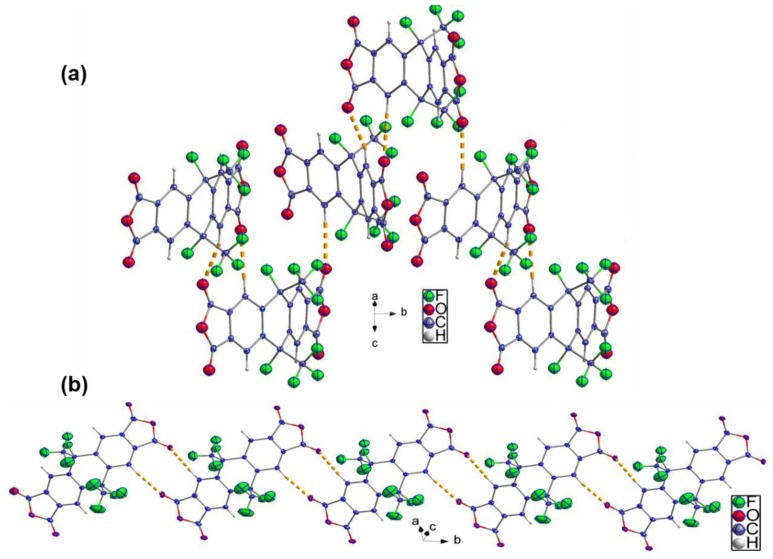
The one-dimensional chain structures of two polymorphs of compound **6** (8FDA), (**a**) zigzag (**6a**), and (**b**) linear (**6b**) chains by C–H…O interactions shown as dashed lines.

For compounds **6a** and **6b**, they are non-solvated, in which the molecules are linked by intermolecular C-H…O interactions into one-dimensional structure along the [0 1 0] axis. In **6a** it appears as wavy-like shape, and in **6b** it looks like more linear (Figure 2a,b). There also exists extensive F…F and C–F…π interactions. The molecules in **6c** are assembled into a two-dimensional layer structure (Figure S22). Because of the incorporation of toluene, no F…F and C–F…π interactions were observed. This indicates that 8FDA with different morphologies may react with the specific di-amine to form PI-1 and PI-2 via the assistance of different intermolecular interactions. Further research of the effect of the crystallization process such as temperature and solvent on the morphologies of 8FDA and its reactivity will be carried out by us in the near future.

### 3.3. The Solubility and WAXD of the Polyimide Films

The solubility of the PI films was tested in various solvents, and the results are summarized in Table 1. Most of the PI films were soluble in typical polar solvents such as *N*,*N*-dimethylacetamide (DMAC) and *N*-methyl-2-pyrrodidon (NMP) at room temperature. The data listed in Table 1 demonstrates that the solubility of films derived from 8FDA is as good as 6FDA and HPMDA for given diamines. It can be explained that the rigid 8FDA monomer owned more linear, semi-alicyclic structures and CF_3_ groups, which disrupted the chain packing and reduced the interchain interaction. It also reduced the crystallinity and endowed good solubility. It is reported that [27,36] highly organo-soluble PI films include some solubility promoting groups (e.g., CF_3_, –O–, and flexible groups) [37,38]. These segments would hinder polymer-polymer interaction, and thus disrupt the chain packing, prevent crystallization, and improve solubility.

Further evidence could be obtained from the wide angle X-ray diffraction (WAXD) measurement. The average inter-chain spacing distance (*d*-spacing) was calculated from the most prominent WAXD peak in the glassy amorphous polymer spectra based on Bragg’s equation: 2*d*sinθ = *n*λ (*n* = 1, λ = 1.54 Å) [15]. From Figure 2b, it was observed that all of the films presented amorphous states. Generally, the polyimide films exhibited d-spacing value in the range of 5.79–6.39 Å. Additionally, the solvent resistance of PIs with the same dianhydride was shown in the decreasing order of TFODA > TFDB, which agreed with the reduced nonplanar steric degree and improved rigidity of mentioned diamines.

### 3.4. Optical Properties of PIs

**Figure 3:**
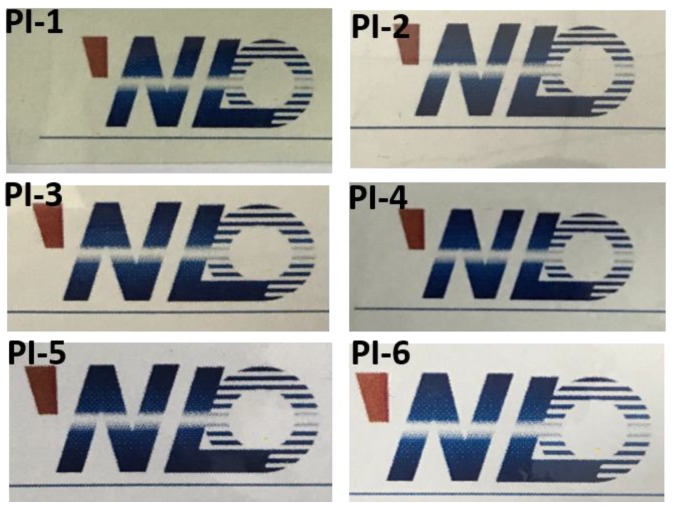
Images of representative 8FDA-, 6FDA-, and HPMDA-based polyimides films.

**Figure 4:**
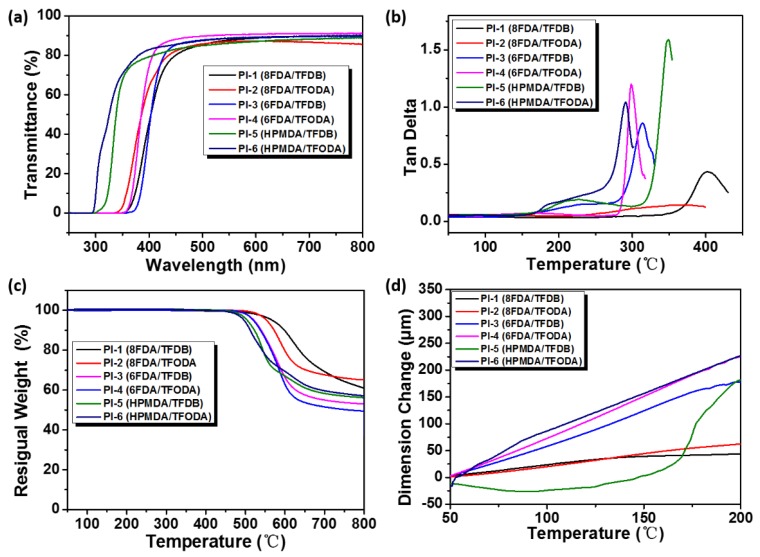
(**a**) The UV–Vis spectra of CPI films; (**b**) the DMA curves of PI films; (**c**) the TGA curves of PI films; and (**d**) the TMA curves of PI films.

Pictures of PI films are shown in Figure 3. The UV-Visible spectra of all films were shown in Figure 4a, and the transmittance at 400 nm (*T*_400_) and cutoff wavelength (λ_0_) were summarized in Table 1, and, according to Figure 4a, 8FDA-based films exhibited *T*_400_ from 46% to 64% and λ_0_ below 345 nm. Additionally, 6FDA series films showed *T*_400_ from 38% to 74% and λ_0_ below 360 nm. Meanwhile, PIs that originated from HPMDA exhibited *T*_400_ from 79% to 83% and λ_0_ below 295 nm. The *T*_400_ of PIs with the same diamines decreased in the order of HPMDA > 6FDA > 8FDA. Although the T_400_ of 8FDA-based PIs was slightly lower than the contrast dianhydrides, it was still better than other reported rigid alicyclic, (Ref. [15] *T*_400_ lower than 10%, Ref. [39] *T*_400_ lower than 20%, Ref. [40] *T*_400_ lower than 15%, and so on) and much better than the traditional ArPIs with the color of deep yellow or brown. Additionally, it can be explained that the existence of 1,4-cyclohexadiene segments can break the conjugation of the polymer chain, which can decrease the charge transfer [29]. In addition, the electron-withdrawing, steric hindrance of pendent CF_3_ groups and fluorine atoms enlarged the average distance of the backbone and induced the separation of the chain backbones, and reduced the inter- and intra-molecular CTC formation that imparted brown color to the polymer as well, which was largely hindered, and optical properties improved [27]. These factors helped 8FDA-based polyimides (PI-1 and PI-2) possess good optical properties.

The transmittance of polyimides derived from the same dianhydride with different diamines showed the tendency of TFODA > TFDB. For instance, PI-3 (8FDA/TFDB) showed *T*_400_ of 43% and λ_0_ of 370 nm, whereas *T*_400_ and λ_0_ values of the PI-2 (8FDA/TFODA) were 71% and 353 nm, respectively. It can be explained that TFODA monomer consists of flexible ether bond, and –CF_3_ groups caused bigger steric hindrance, smaller interchain interaction, and weakened CTC than TFDB [41].

The cutoff wavelength (λ_0_) of a polyimide film was another parameter of the optical transparency; herein, the cutoff was considered to be the wavelength at which the transmittance became less than 1% [29], and the dada is summarized in Table 2. As the transmittance of PI-x improved, the cutoff wavelength shortened [33]. For example, PI-2 with *T*_400_ of 64% and λ_0_ of 344 nm exhibited a shorter cutoff wavelength than PI-1with *T*_400_ of 46% and λ_0_ of 358 nm. Therefore, the optical properties of 8FDA-based PIs met the applicable criterion for some advanced optoelectronics.

### 3.5. Thermal Properties of PIs

The thermal decomposition and stability properties of the films of PIs were evaluated by thermogravimetric analysis (TGA), dynamic mechanical analysis (DMA), and thermal mechanical analysis (TMA). All data were obtained from the PIs films, and are shown in Table 2. According to the DMA curve in Figure 4b, it was found that 8FDA-based PIs exhibited higher *T*_g_ values than the corresponding 6FDA-based and HPMDA-based PIs films. The *T*_g_ values of PI-1 and PI-2 were in the range of 369–401 °C, while the *T*_g_ of the contrast PIs (PI-3 to PI-6) were in the range of 292–348 °C. The *T*_g_ of PI-1 is 401 °C, which is 86 °C higher than that of PI-3. This can be attributed to the rigid structure of 8FDA monomer. According to the single crystal structure, 8FDA has 1,4-cyclohexadiene as the bridge between two phenyl anhydrides. The planarity of 8FDA is much better than that of 6FDA and HPMDA, with a bridge of quaternary carbon between two phenyl anhydrides and alicyclic structure. The linear structure, rigidity, and inter-chain interaction of 8FDA-based PIs were stronger than the PIs based on 6FDA and HPMDA [42]. According to the literature, the *T*_g_ of polyimides were dictated by the chain rigidity, as well as the inter- and intra-molecular interactions that originated from the CTC between the electron-withdrawing dianhydride residue and the electron-donating diamine residue [28]. For a given dianhydride, the *T*_g_ value of PIs were in the order of TFDB > TFODA. Because the rigidity of TFODA, which consisted of flexible ether, the bond was lower than TFDB.

As shown in Table 2 and Figure 4c, no significant weight loss was detected until the temperature was as high as 460 °C. The 1% weight loss temperature (*T*_d1_) and the char yield of these polyimides ranged from 462 to 518 °C and 50%–64%, respectively. 8FDA-based PIs exhibited higher *T*_d1_ more values than the corresponding 6FDA and HPMDA films. As mentioned above, multi-substituted and rigid semi-alicyclic 1,4-cyclohexadiene in 8FDA featured more rigid structure, which enhanced the inter-chain interaction and accumulation degree [43], as well as increased their thermal resistance.

The 5% weight loss temperature (*T*_d5_) of PI-x in N_2_ stayed in the range of 497–557 °C. The curves in Figure 4c indicated that the thermal stability of PI-x originated from the same dianhydride decreased in the order of TFDB and TFODA. It can be accounted for that TFDB-based PIs possessed higher rigidity and showed good thermal stability. The TFODA monomer was bridged by ether bond, and the bond energy of C–O is 326 KJ mol^−1^, which is the lowest one among all the bonds in the backbone. When it was heated, the PIs based on TFODA were more prone to fracture; they thus decreased the thermal resistance of TFODA-based PIs.

The TMA results are shown in Table 2 and Figure 4d. The CTE values of PI-1 and PI-2 derived from 8FDA were 14 to 18 ppm K^−1^, respectively, while the contrast PIs based on 6FDA and HPMDA ranged from 44 to 63 ppm K^−1^. Compared with 6FDA and HPMDA, it is the bent-shaped rigid dianhydride moiety containing cis-type 1,4-cyclohexadiene skeleton that suppress CTE. As described in the literature [44], coefficient of thermal expansion (CTE) is divided into the lateral (in-plane) CTE of polyimide films and the vertical (out-of-plane) CTE. Additionally, the vertical CTE values were different from lateral CTE. In this work, the CTE data were the lateral CTE data and were measured using a thermal mechanical analyzer (TA Instruments, New Castle, PA, USA) in expansion mode. Ando’s [45] group reported the relationship between the molecular orientation and CTEs parallel and perpendicular to the film plane. They evaluated these CTE values separately and also exact CVE value by means of not only conventional TMA measurement but also an optical interferometric method in near-IR region, respectively. They found a good linear relationship between anisotropy in CTE and molecular orientation. If the intrinsic birefringence values, Δn_0_, are same among the target samples, one can directly compare the birefringence, although Δn_0_ value depends on the density and also optical polarizability tensor components. As shown in Figure S23, 8FDA-based PI-1 and PI-2 possessed biggest birefringence (0.129 for PI-1, 0.123 for PI-2); it was reported [46] that birefringence data ≥0.1 represents a criterion in which aromatic PI chains are highly aligned parallel to the film plane, so that lateral CTE values of 8FDA-based PIs were lower than the contrast PI-3-PI-6 (from 0.016 to 0.066), which originated from 6FDA and HPMDA. Additionally, in the OLED display area, dimensional stability is more concerned about lateral CTE. As depicted in the reference, the CVE of aromatic PIs decreased with increasing the density. WAXD data revealed longer intermolecular distance of PI-1 and PI-2, that is, lower density, so that the CVE of PI-1 and PI-2 was bigger than contrast PIs. It can be explained that according to the single crystal diffraction test, 8FDA has more linear and semi-alicyclic modified structure, which enlarged the average distance of 8FDA-based polyimides and led to lower density and increased the CVE.

Compared with the CTE value of polyimide films derived from the more rigid dianhydride (PMDA and NTDA) with rigid diamines and summarized in Table S2, it was found that, for a given dianhydride, with increasing the rigidity of diamine, the CTE value decreased [47]. It can be explained that the PMDA [46,48] and the NTDA [49,50] monomer had more rigid and linear structure, while endowed rigid dianhydride-based polyimides have better in-plane orientation in CTE than 8FDA-based polyimide. For instance, CTE of PI-1 (8FDA/TFDB) was 14 ppm K^−1^, while the CTE of CPI-1(NTDA/APAB) and CPI-3 (PMDA/APAB) was 3.0 and 2.0 ppm K^−1^, respectively, so that the orientation, density, and the structure of PIs affected the CTE value together. The results revealed that 8FDA is a useful dianhydride monomer that provides unique PIs possessing lower CTE [15,28].

### 3.6. Mechanical Properties

**Figure 5:**
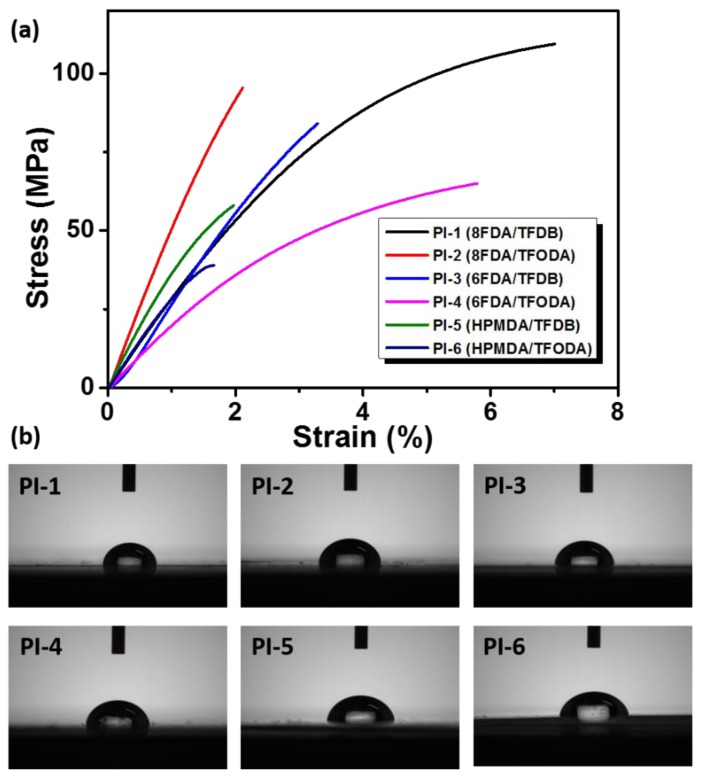
(**a**) The strain-stress curves of PI films and (**b**) representative images of contact angles of the polyimide films.

Mechanical properties of the PIs are summarized in Table 2. The stress-strain curves are shown in Figure 5a. PI-1 and PI-2 showed the tensile strengths at break of 96.0–113.0 MPa, the elongation at break of 2.1–7.0%, and the tensile modulus of 1.2–4.5 GPa. As a comparison, PI-3 to PI-6 showed lower tensile strengths at break of 39.0–84.0 MPa, comparable elongation at break of 2.7–5.8%, and lower tensile modulus of 1.1–1.8 GPa. Figure 5a depicts that PI-1 and PI-2 exhibited better mechanical properties than contrast PI-x (6FDA and HPMDA series). It can be explained that molecular structure of 8FDA, which was bridged by multi-substitute 1,4-cyclohexadiene segment, was more planar and regular than 6FDA and HPMDA. These characters made 8FDA-based PIs exhibit stronger rigidity and closer accumulation. The structure characters of 8FDA enhanced its mechanical properties. Both series followed the tendency based on the same dianhydride in the decreasing order of TFDB and TFODA in terms of tensile strengths and tensile modulus. It should be noted that the tensile stress and tensile module enhanced as the rigidity of the PI’s main chain increased [51]. Thus, the results of tensile curves demonstrated that by incorporating the rigid semi-alicyclic segment in the backbone of the polyimide, the tensile strengths, the tensile modulus, and the elongation at break were significantly enhanced.

### 3.7. Surface Properties of the PIs

Figure 5b described the contours of the droplet on the PI-x surface. Additionally, the contact angle was summarized in Table 1. As shown in Table 1 and Figure 5b, all of the contact angles against water of the PIs were above 70° [38], in the range of 82.0°–95.0°, which demonstrated that all the surfaces of PI-x were hydrophobic. The increased hydrophobicity of 8FDA-based PIs (θ values range from 92°–95.0°) can be attributed to its steric effects, semi-alicyclic segments, and higher content of fluorine atoms. Compared with 6FDA- and HPMDA-derived PI-x, 8FDA-based PI-x, being more rigid and planar, had higher regularity and packing degree, which increased their hydrophobicity. It has been reported that alicyclic segments in the backbone can improve the hydrophobicity of the PIs [33]. Meanwhile, the trifluoromethyl groups and fluorine atoms made the migration of the fluorocarbon chain to the film surface and fluorine enrichment at the surface [52], which reduced the surface tension and made the surface become more hydrophobic.

PIs usually showed higher moisture uptakes than the hydrocarbon polymers because of the hydrophilic imide groups [53]. It should be noted that structures of the PIs played an important role in the moisture absorption. It can be seen in Table 1 that all the PIs exhibited low water uptakes. The better hydrophobicity of 8FDA-based PIs led to the low affinity for water. Additionally, the trifluoromethyl groups and fluorine atoms possessed water repelling features, which inhibited the absorption of moisture molecules on the surface of the fluorinated polymer films [54].

It is reported that alicyclic segments of the main chain can decrease the moisture uptakes of PIs. Therefore, it was found that PIs based on 8FDA possessed the lowest water barrier properties. Besides, PI-x exhibited lower water uptake than traditional aromatic polyimide. These results demonstrated that polyimides containing rigid, semi-alicyclic segments and fluorine-contained groups showed greater practical reliability with regard to withstanding long-term humid working environment for optoelectronics and microelectronics application.

**Figure 6:**
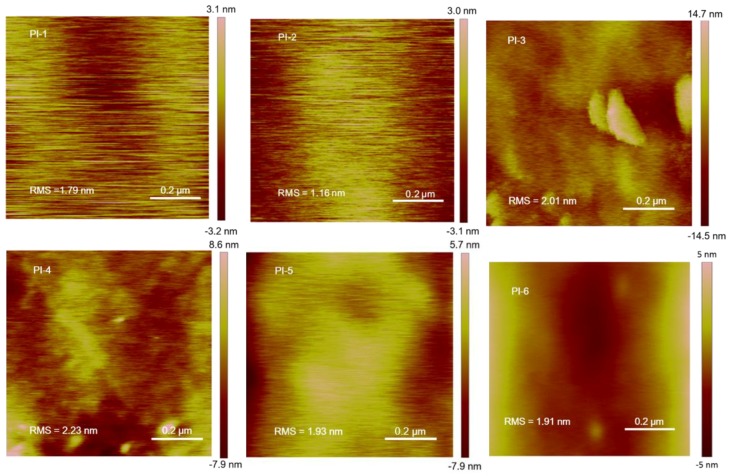
The AFM images of PI-x.

Atomic Force Microscope (AFM) was used to characterize the surface morphologies and roughness of polymer films. AFM images are shown in Figure 6. Additionally, the root mean square (RMS) roughness is listed in Table 1. Normally, RMS strongly interrelated with the degrees of order or disorder in the grain structure distribution and associated with the shape-size complexity [55]. Therefore, PI-x originated from 8FDA, which possessed more neat main chains and exhibited lower RMS. For instance, RMS value of PI-1 (8FDA/TFDB) is 1.16 nm, and PI-2 (8FDA/TFODA) is 1.79 nm. Additionally, all the PIs owned low RMS, which indicated that PIs can be used as flexible substrate.

## 4. Conclusions

In conclusion, a polymorphic dianhydride 8FDA and a transparent polyimide based on 8FDA were reported. The geometric configuration transition during the synthesis of 8FDA was traced using single crystal X-ray diffraction of the intermediate compounds. Compared with the widely used dianhydride 6FDA and HPMDA, 8FDA-based transparent PI films showed much better thermal and dimensional stabilities. The new building block for transparent PIs is a promising key monomer toward the substrate for flexible OLEDs display.

## Figures and Tables

**Table 1:** The solubility, *d*-spacing value, and optical properties of PI films.

Polymer Code	Dianhydride	Diamine	Solubility ^a^								WAXD ^b^		Surface Properties		
			THF	EA	Toluene	CH_2_Cl_2_	m-Cresol	DMAc	NMP	DMF	2θ (°)	*d*-Spacing (Å)	Water Absorbtion (%) ^c^	Contact Angle (°) ^d^	RMS (nm)
PI-1	8FDA	TFDB	+s	+s	-	+-	+s	+s	+s	+s	14.01	6.31	0.85	95	1.79
PI-2	8FDA	TFODA	+s	+s	-	+	+	+	+	+	13.84	6.38	1.06	92	1.16
PI-3	6FDA	TFDB	+-	+-	-	+-	+	+s	+	+s	14.38	6.20	1.21	92	2.01
PI-4	6FDA	TFODA	+	+-	-	+-	+	+s	+	+s	14.28	6.26	1.64	90	2.23
PI-5	HPMDA	TFDB	-	-	-	-	+	+s	+	+s	15.10	5.79	1.21	84	1.93
PI-6	HPMDA	TFODA	-	-	-	-	+	+	+	+	15.28	5.86	1.33	82	1.91

^a^ Qualitative solubility measured with 100 mg of the polymer in 2 mL of solvent. +, soluble at room temperature; +s, soluble after heating; +-, slightly soluble after heating; -, insoluble; ^b^ The most prominent WAXD peak in the amorpous glassy polymer spectra was adopted to calculate the *d*-spacing value according to Bragg’s equation: 2*d*sinθ = *n*λ (*n* = 1, λ = 1.54 Å); ^c^ Water absorption (%) = ((*W* − *W*_0_)/*W*_0_) × 100%, in which *W* is the weight of polymer sample after being immersed in water for 96 h at room temperature and *W*_0_ the weight of polymer sample after being dried in vacuum at 100 °C for 8 h. ^d^ Equilibrium contact angle was measured at ambient temperature using double distilled water as solvent for a time period of 120 s depending on the stability of the drop, and the data were the average value of 10 experiments.

**Table 2:** The thermal resistant, mechanical, optical, and surface properties of PI films.

Polymer	Dianhydride	Dianmine	Thermal Properties					Mechanical Properties ^a^			Optical Properties	
			*T*_g_ (°C) DMA	*T*_d1_ (°C)	*T*_d5_ (°C)	Char Yield (%) ^b^	CTE (ppm K^−1^) ^c^	*T*_s_ (MPa)	*E*_b_ (%)	*T*_m_ (GPa)	*T*_400_^d^ (%)	λ_0_^e^ (nm)
PI-1	8FDA	TFDB	401	518	557	64	14	113	7.0	1.2	46	358
PI-2	8FDA	TFODA	369	492	550	60	18	96	2.1	4.5	64	344
PI-3	6FDA	TFDB	315	488	521	53	49	84	3.3	2.6	43	370
PI-4	6FDA	TFODA	300	490	520	50	63	65	5.8	1.1	71	353
PI-5	HPMDA	TFDB	292	473	501	56	44	71	4.0	1.8	79	304
PI-6	HPMDA	TFODA	348	462	497	57	64	39	2.7	1.8	83	296

^a^*T*_s_, tensile strength; *E*_b_, elongation at break; *T*_m_, tensile modulus; ^b^ residual weight percentage at 800 °C in N_2_; ^c^ coefficient of thermal expansion along the X-Y direction, measured in the range of 50–200 °C at a heating rate of 5 K min^−1^; ^d^ transmittance at 400 nm; ^e^ UV-vis cutoff wavelength.

## Supplementary Materials

The following are available online at http://www.mdpi.com/2073-4360/10/5/546/s1.
