# Supplementary material for: High Performance Soluble Polyimides from Ladder-Type Fluorinated Dianhydride with Polymorphism
Source: Polymers (Basel). 2018 May 18;10(5):546. doi: 10.3390/polym10050546 (PMC6415444; doi:10.3390/polym10050546)
Supplement: Supplementary file 1 [file polymers-10-00546-s001.pdf]

Supplementary Materials

High Performance Soluble Polyimides from Ladder-Type Fluorinated Dianhydride with Polymorphism

Fu Li [1], Jikang Liu [1], Xiangfu Liu [1], Yao Wang [1], Xiang Gao [2], Xianggao Meng [3,*] and Guoli Tu [1,*]

1 Wuhan National Research Center for Optoelectronics, Huazhong University of Science and Technology, Wuhan 430074, China; lifu@hust.edu.cn (F.L.); liujikang@hust.edu.cn (J.L.); xfliu@hust.edu.cn (X.L.); m201772826@hust.edu.cn (Y.W.)
2 School of Materials Science and Engineering, Wuhan Institute of Technology, Wuhan 403052, China; gaoxiang@hust.edu.cn
3 Key Laboratory of Pesticide and Chemical Biology of the Ministry of Education, College of Chemistry, Central China Normal University, Wuhan 430079, China
* Correspondence: xianggao_meng@126.com (X.M.); tgl@hust.edu.cn (G.T.)

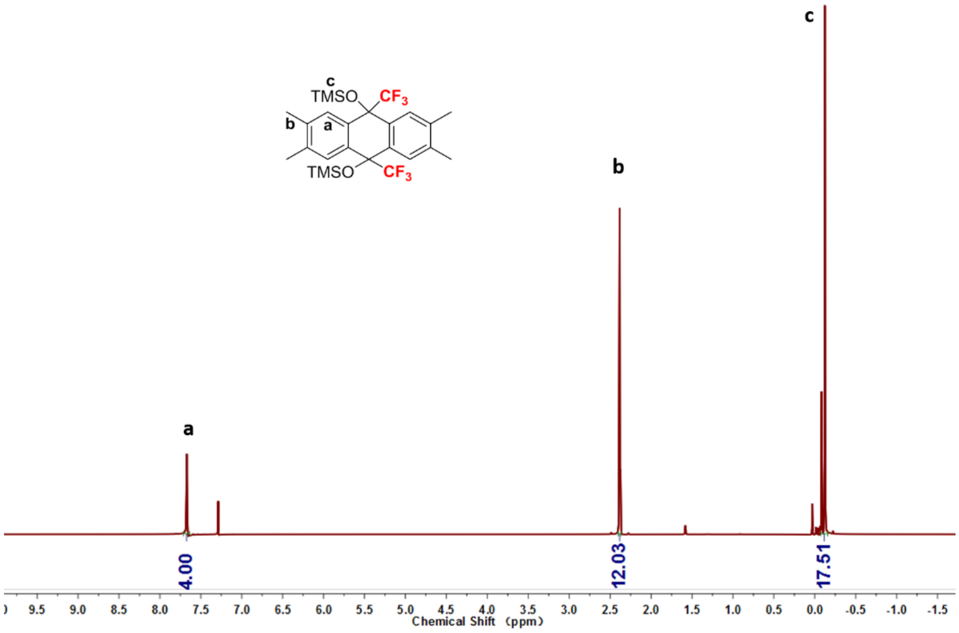

Figure S1. The ^{1}H NMR spectrum of compound **2**.

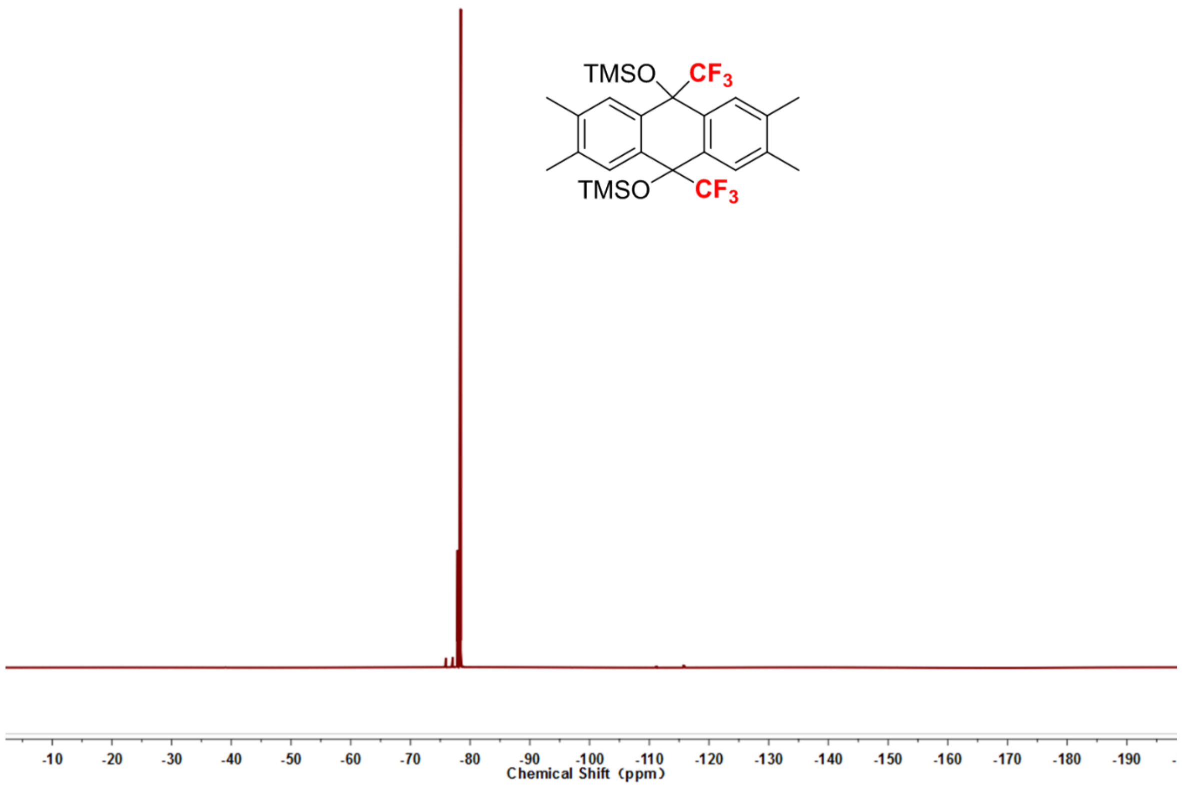

Figure S2. The ^{19}F NMR spectrum of compound **2**.

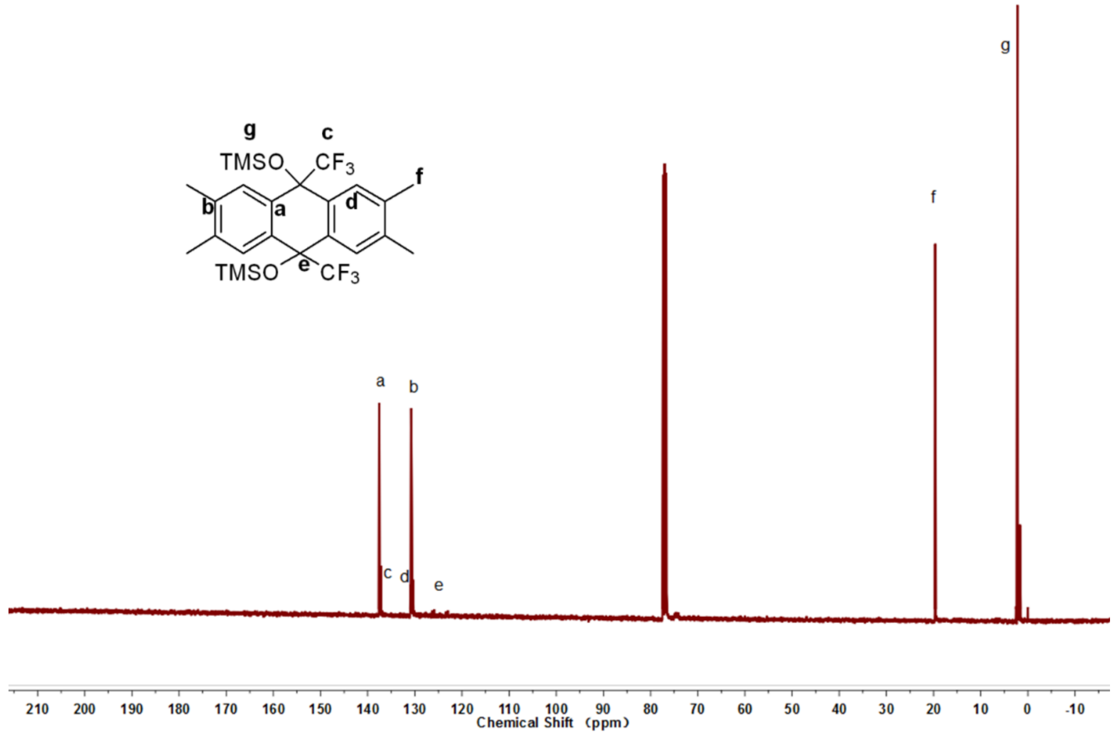

Figure S3. The ^{13}C NMR spectrum of compound **2**.

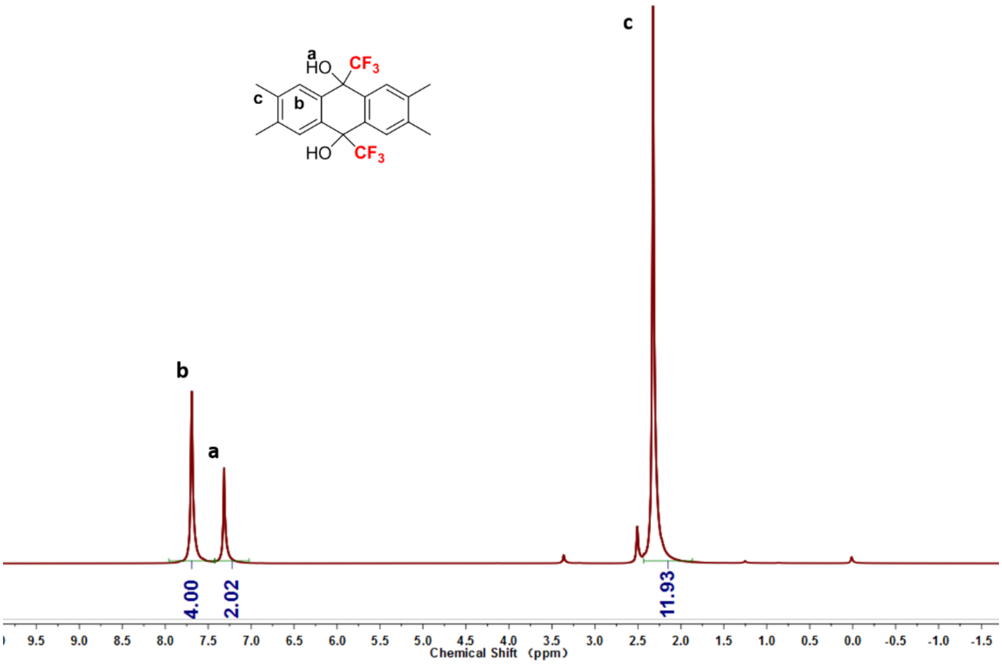

Figure S4. The ^{1}H NMR spectrum of compound **3**.

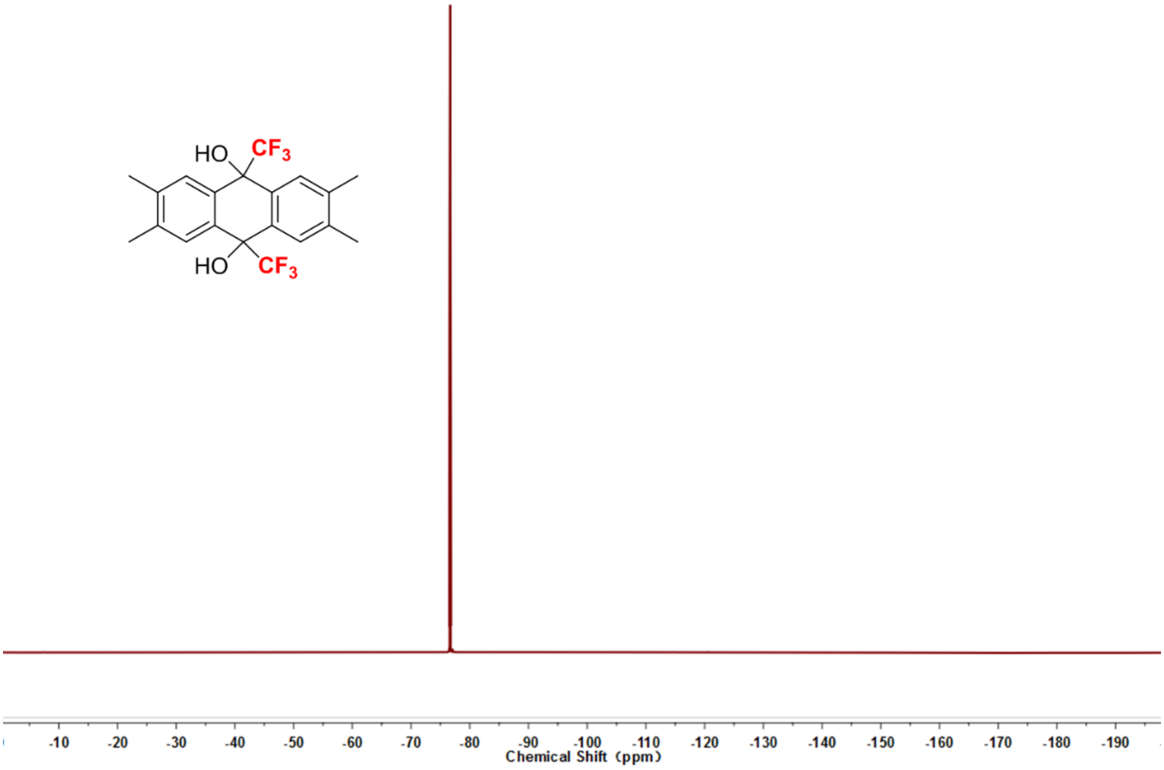

Figure S5. The ^{19}F NMR spectrum of compound **3**.

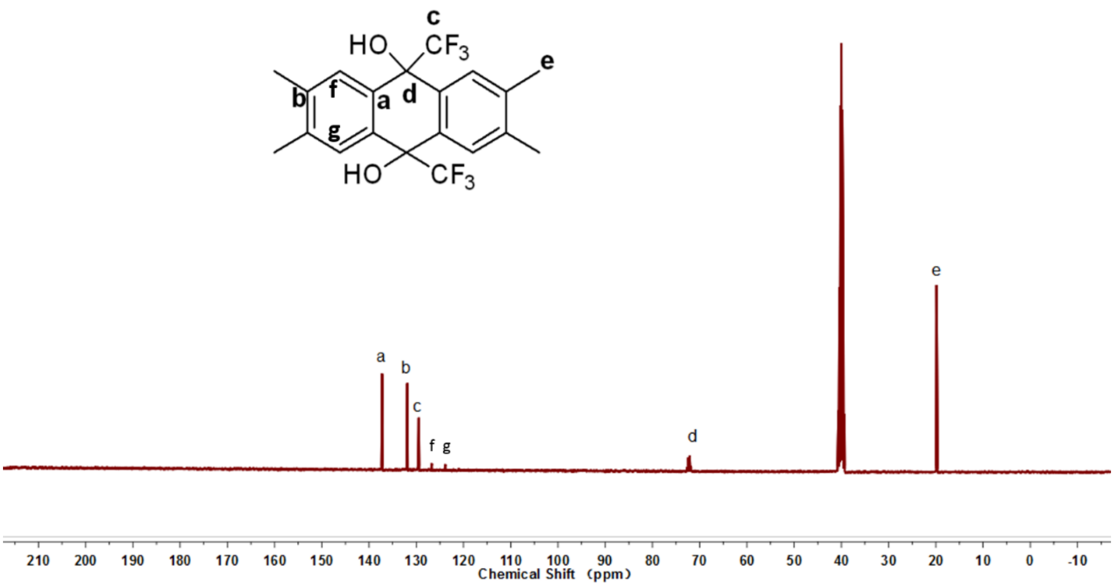

Figure S6. The ^{13}C NMR spectrum of compound **3**.

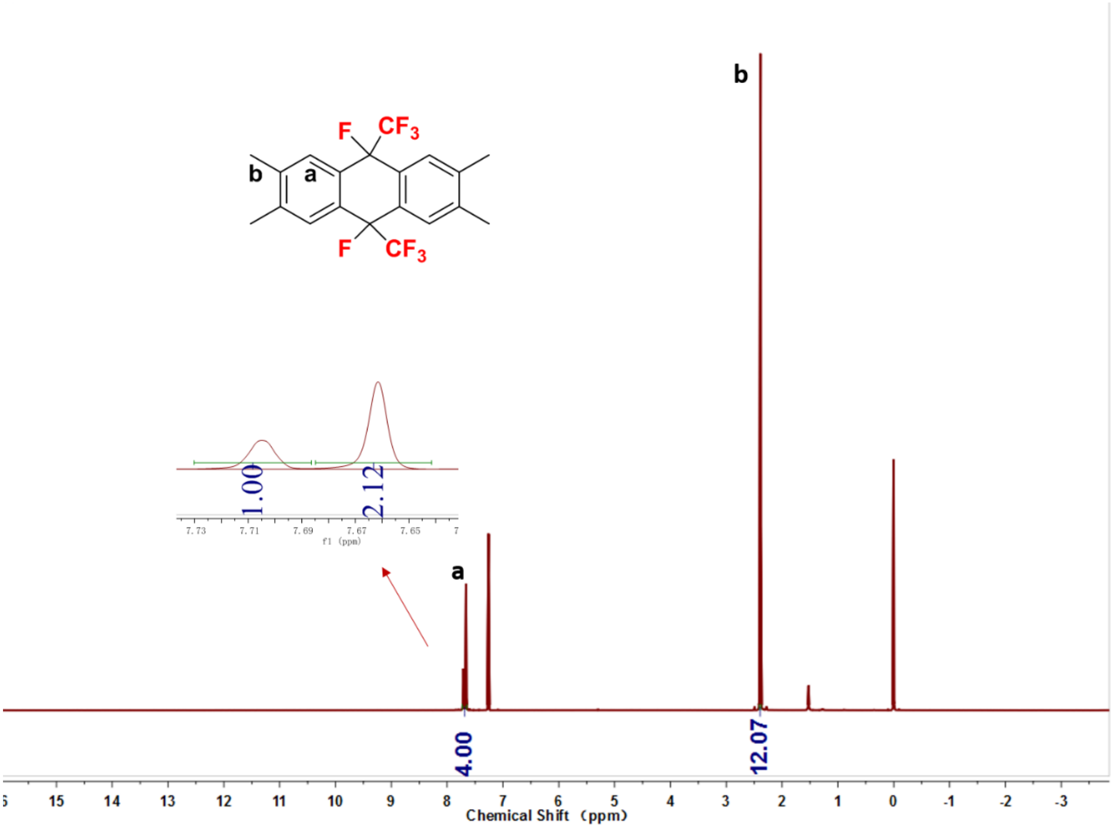

Figure S7. The ^{1}H NMR spectrum of compound **4**.

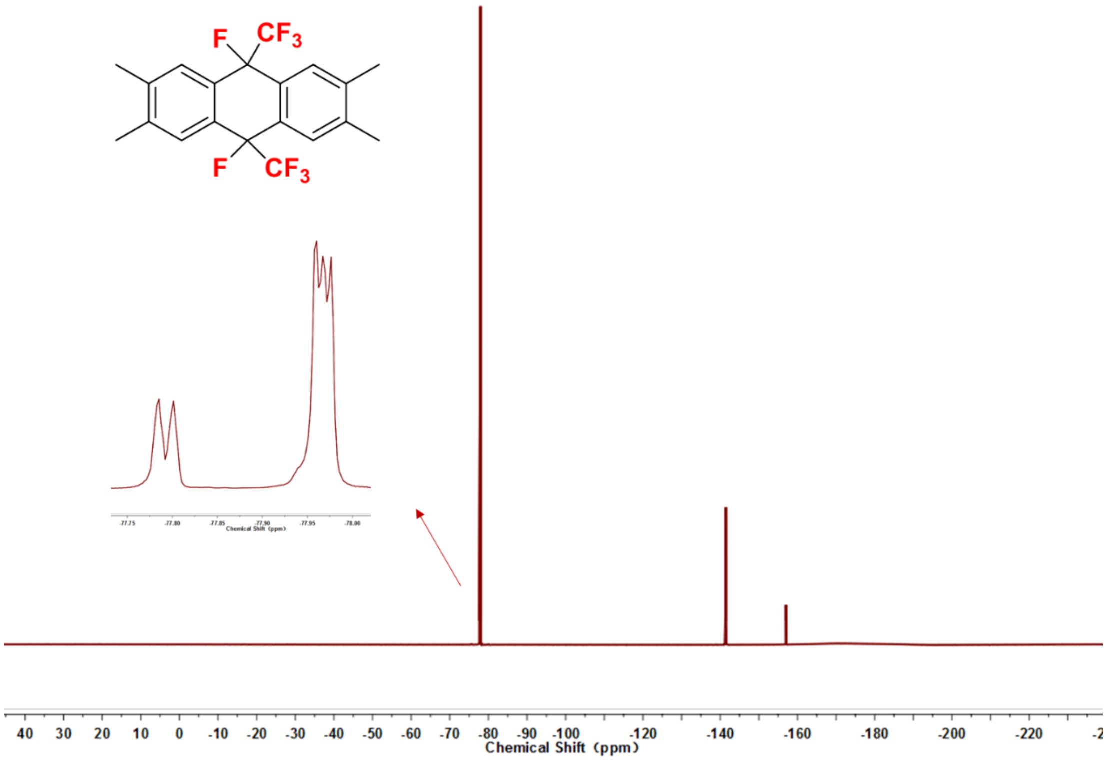

Figure S8. The ^{19}F NMR spectrum of compound **4**.

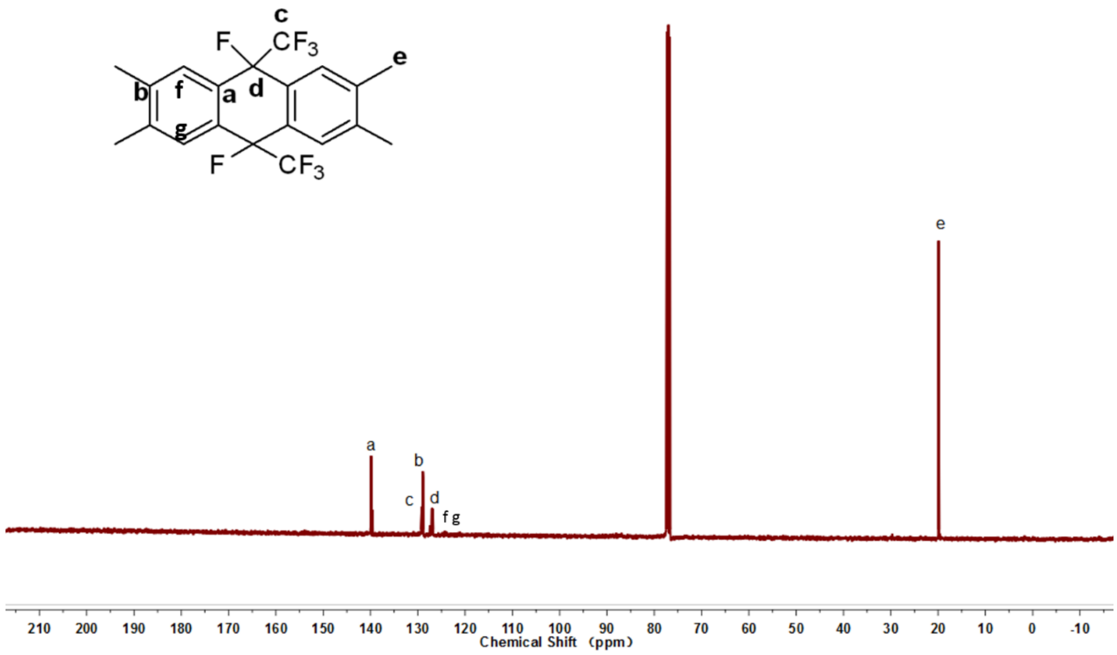

Figure S9. The ^{13}C NMR spectrum of compound **4**.

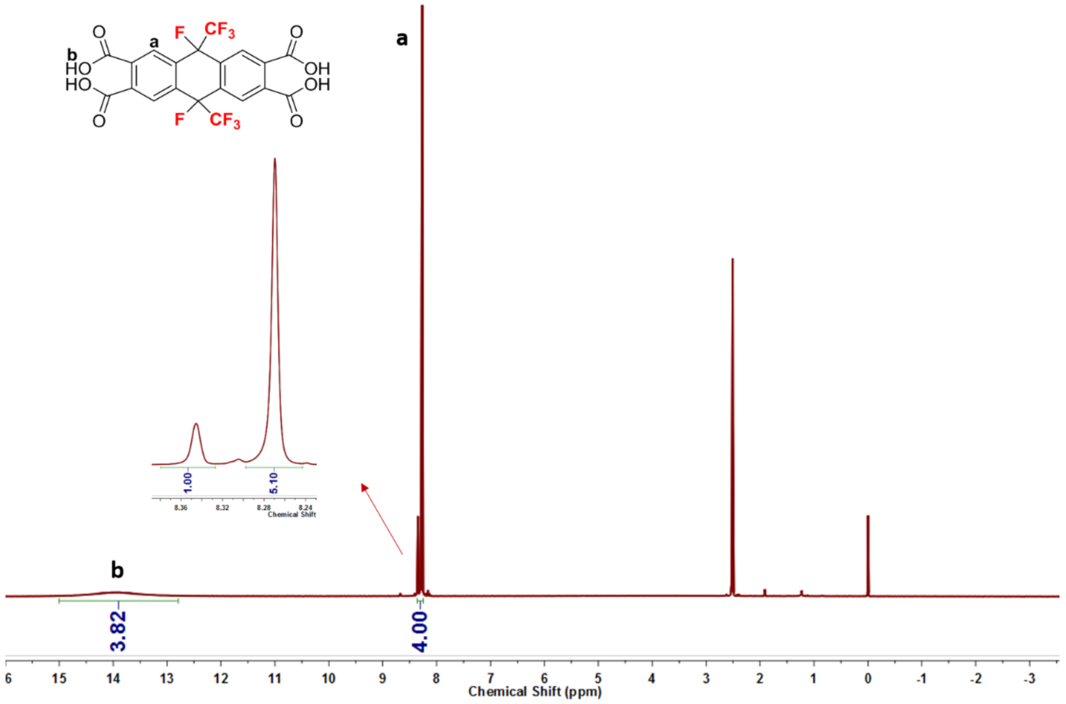

Figure S10. The ^{1}H NMR spectrum of compound **5**.

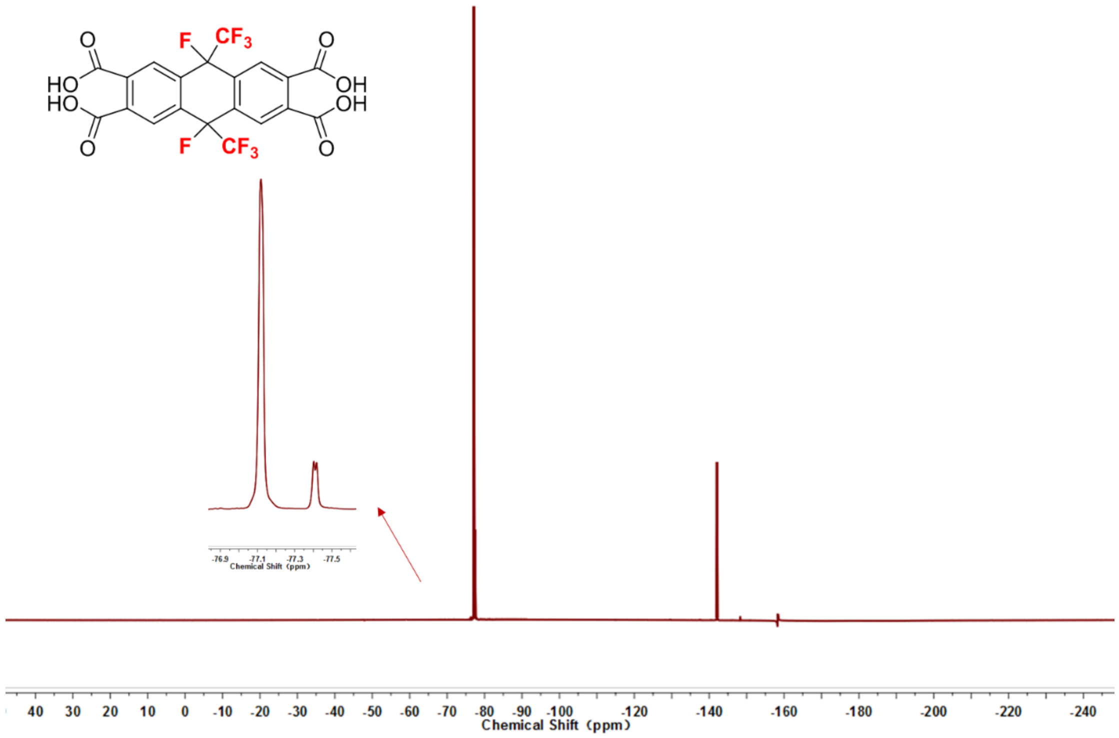

Figure S11. The ^{19}F NMR spectrum of compound **5**.

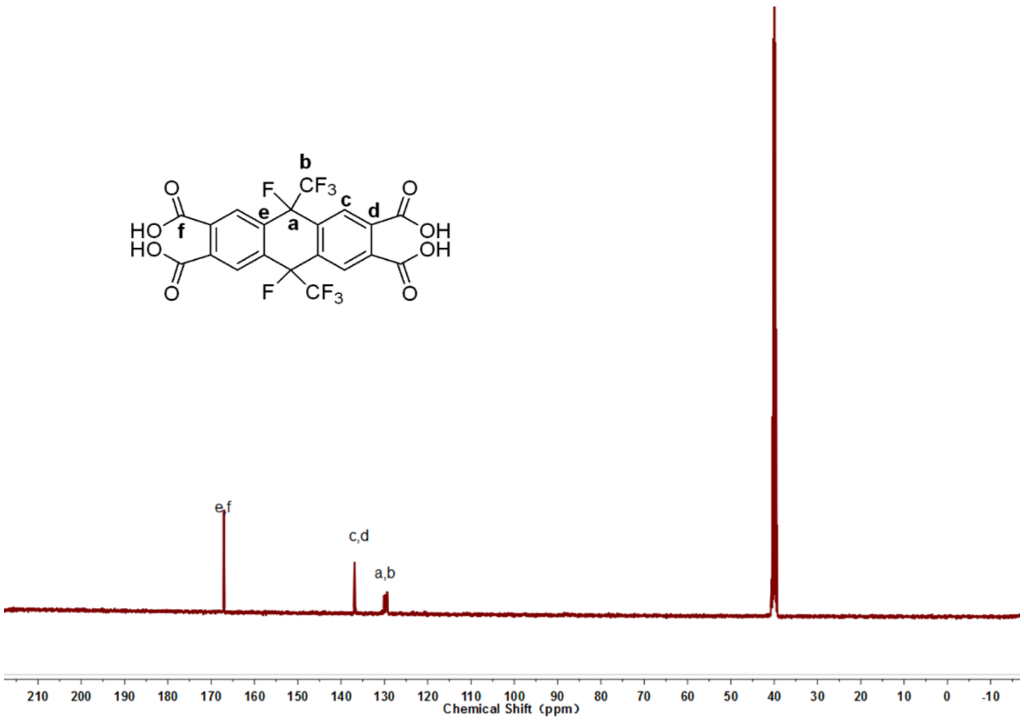

Figure S12. The ^{13}C NMR spectrum of compound **5**.

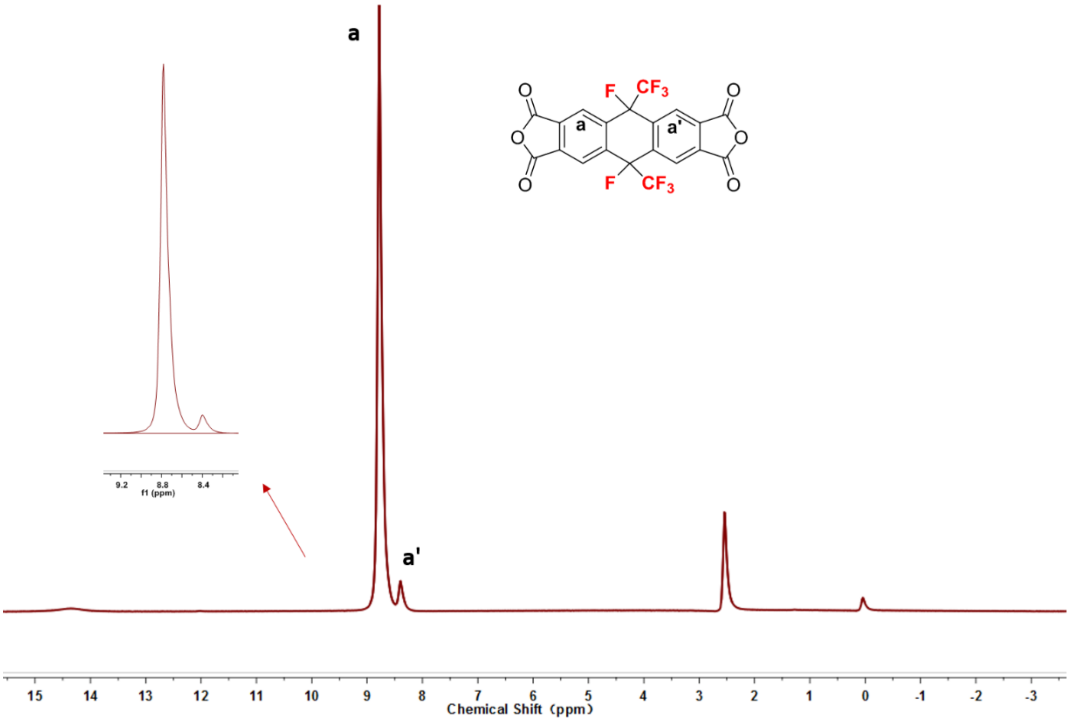

Figure S13. The ^{1}H NMR spectrum of compound **6** (8FDA).

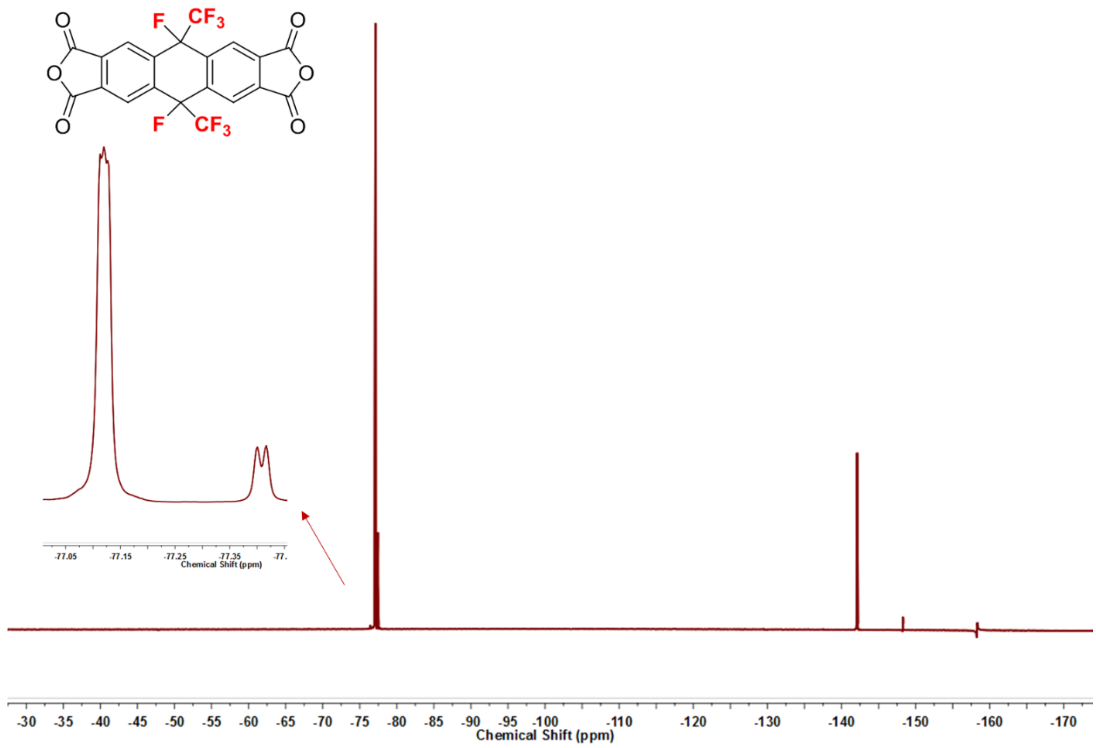

Figure S14. The ^{19}F NMR spectrum of compound **6** (8FDA).

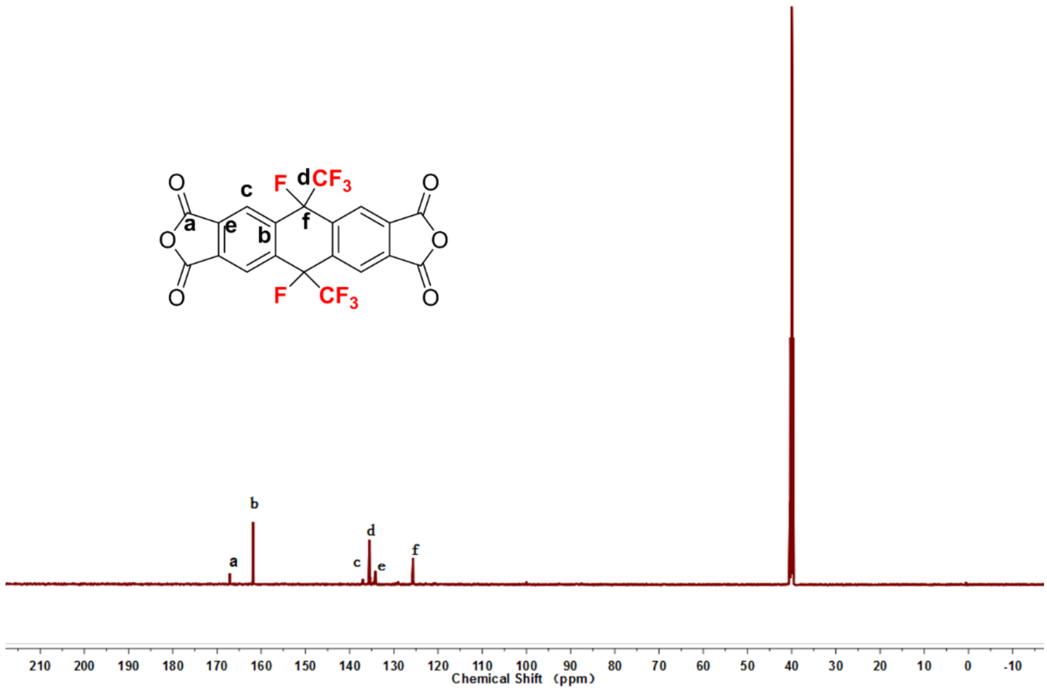

Figure S15. The ^{13}C NMR spectrum of compound **6** (8FDA).

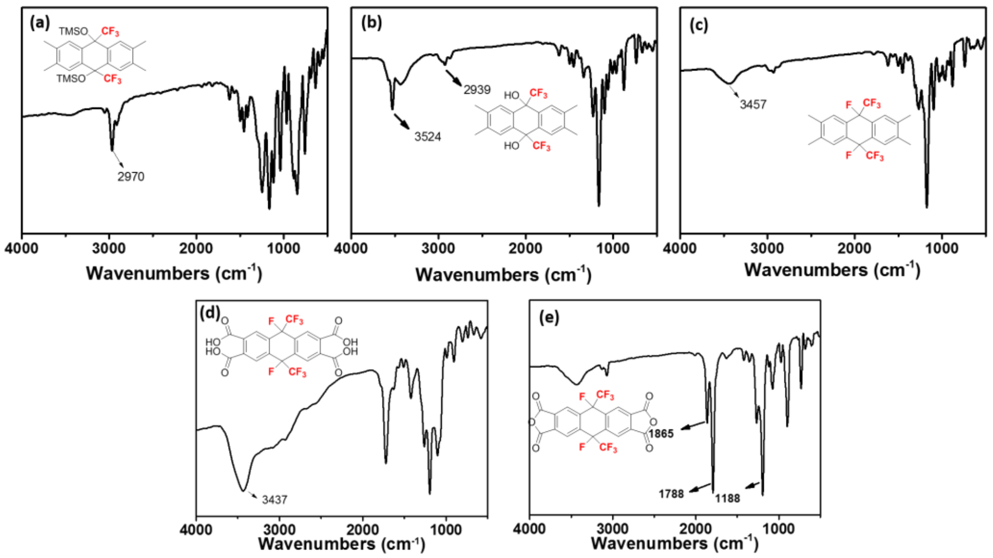

Figure S16. (a) FT-IR spectra of compound **2**. (b) FT-IR spectra of compound **3**. (c) FT-IR spectra of compound **4**. (d) FT-IR spectra of compound **5**. (e) FT-IR spectra of **6** (8FDA).

Mechanism analysis

The changing of geometric configuration happened in Step 3 [1]. The possible mechanism was described in Scheme S1. Two hydroxyl groups in compound 2 will react with two molecules of DAST to form the intermediate 1. Intermediate 1 was decomposed into a carbenium-containing intermediate 2 and intermediate 3. Then the intermediate 3 will further decomposed into fluorine anions (F-). Then the obtained F- will attack the carbenium ion (position C1) from the less sterically hindered side to form intermediate 4. The sulfur-containing group on the intermediate 4 decomposed into another C+ containing intermediate 5. Then F- attacked C+ (position C2) from less steric obstructed side of C1. Finally, two trifluoromethyl groups appeared on the same side of the benzene ring plane [2,3].

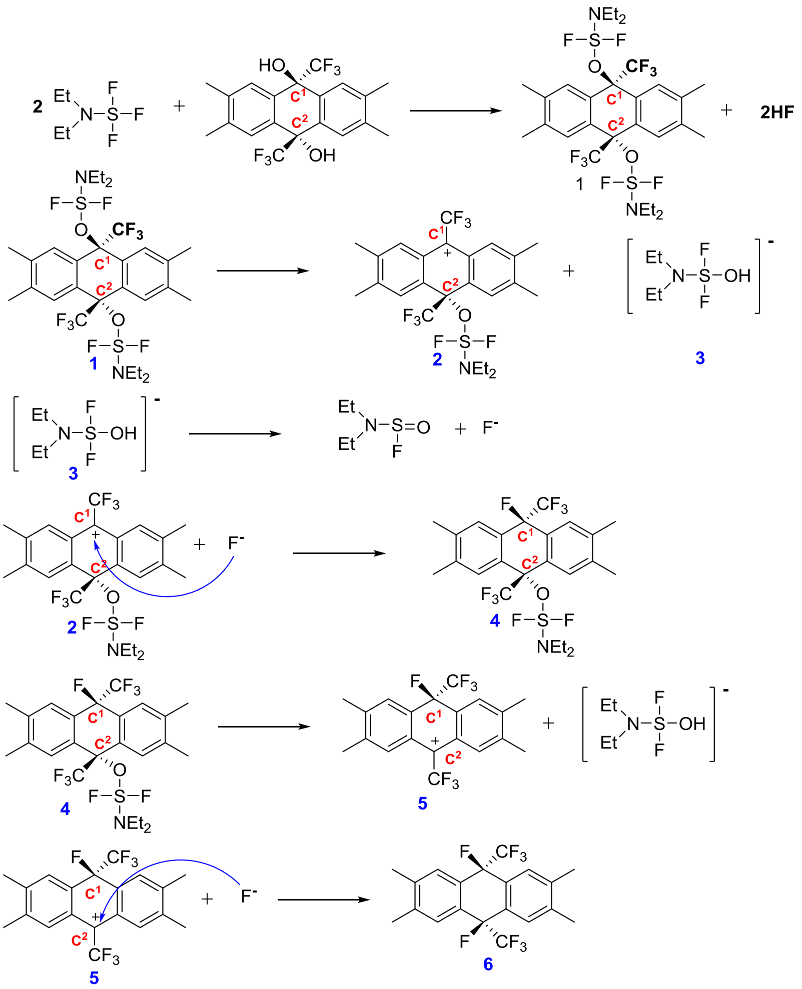

Scheme S1. Suggested mechanism for formation of 8FDA.

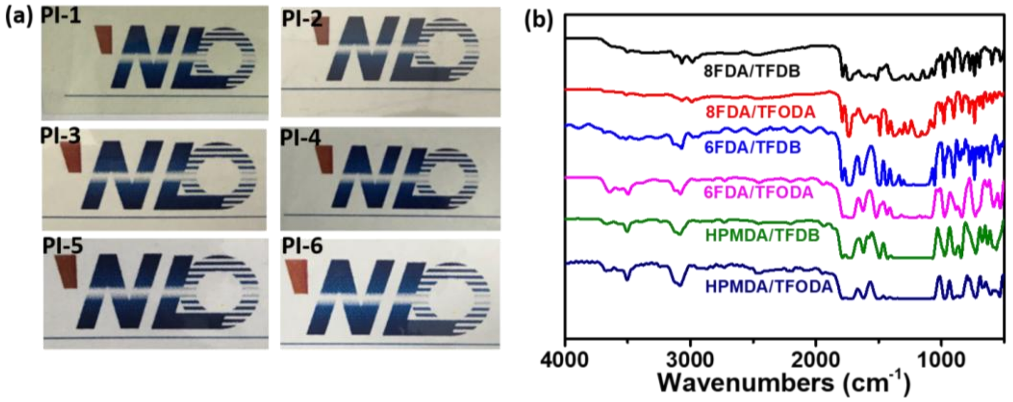

Figure S17. (a)The pictures of PI films. (b) The FT-IR spectra of PIs.

Table S1. The crystal dates of compound **2**, **3**, **4**, **6a**, **6b**, **6c**.

	**2**	**3**	**4**	**6a**	**6b**	**6c**
Empirical formula	$C_{26}H_{34}F_6O_2Si_2$	$C_{20}H_{18}F_6O_2$	$C_{20}H_{15}F_8$	$C_{20}H_4F_8O_6$	$C_{20}H_4F_8O_6$	$C_{34}H_{20}F_8O_6$
CCDC number	1830504	1830505	1830506	1830507	1830508	1830509
Formula weight	548.71	404.34	407.32	492.23	492.23	676.5
Temperature	100(2) K	295(2) K	295(2) K	297(2) K	297(2) K	100(2) K
Wavelength	1.54178 Å	1.54178 Å	1.54178 Å	0.71073 Å	1.54178 Å	1.54184 Å
Crystal system	Triclinic	Tetragonal	Monoclinic	Monoclinic	Monoclinic	Monoclinic
Space group	*P*-1	$I4_1/a$	*P*2/c	$P2_1/c$	$P2_1/c$	$P2_1/c$
Unit cell dimensions (Å)	a = 8.8778(1)	a = 17.5981(4)	a = 7.6688(5)	a = 13.671(5)	a = 9.3165(1)	a = 16.1012(3)
	b = 8.9459(1)	b = 17.5981(4)	b = 8.6511(5) Å	b = 14.842(6)	b = 10.4994(1)	b = 10.5322(2)
	c = 10.1723(1)	c = 11.7161(4)	c = 27.2240(16)	c = 17.277(6)	c = 19.6924(2)	c = 18.1230(4)
Volume (Å^3)	720.378(15)	3628.40(17)	1803.26(19)	3500(2)	1775.03(3)	2855.56(10)
Z	1	8	4	8	4	4
Density calculated (Mg/m^3)	1.265	1.480	1.500	1.869	1.842	1.574
Absorption coefficient (mm^{-1})	1.651	1.192	1.285	0.192	1.715	1.244
F(000)	288	1664	828	1952	976	1376

Crystal size (mm^3)	0.120 × 0.100 × 0.100	0.05 × 0.03 × 0.02	0.080 × 0.040 × 0.030	0.200 × 0.200 × 0.150	0.20 × 0.10 × 0.10	0.150 × 0.120 × 0.100
Theta range for data collection	4.872 to 73.75°.	4.53 to 47.28°.	3.252 to 65.00°.	1.492 to 24.95°.	4.866 to 73.92°.	4.953 to 74.03°.
Index ranges	−11<=h<=10, −11<=k<=8, −10<=l<=12	-16<=h<=16, −16<=k<=16, −11<=l<=11	−8<=h<=7, −10<=k<=10, −31<=l<=31	−16<=h<=15, −16<=k<=17, −20<=l<=20	−11<=h<=11, −11<=k<=13, −24<=l<=21	−20<=h<=19, −6<=k<=12, −22<=l<=22
Reflections collected	6449	21041	19854	23371	18616	31532
Independent reflections	2785 [R_{int} = 0.012]	820 [R_{int} = 0.023]	2998 [R_{int} = 0.062]	6067 [R_{int} = 0.045]	3569 [R_{int} = 0.029]	5713 [R_{int} = 0.102]
Completeness	99.10%	99.50%	97.80%	98.90%	100.00%	100.00%
Data / restraints / parameters	2785 / 0 / 169	820 / 1 / 133	2998 / 0 / 258	6067 / 0 / 613	3569 / 0 / 308	5713 / 0 / 435
Goodness-of-fit on F^2	1.081	1.06	1.159	1.027	1.039	1.051
Final R indices [I>2σ(I)]	R1 = 0.0361, wR2 = 0.1010	R1 = 0.0279, wR2 = 0.0713	R1 = 0.0791, wR2 = 0.2242	R1 = 0.0431, wR2 = 0.1075	R1 = 0.0322, wR2 = 0.0898	R1 = 0.0471, wR2 = 0.1296
R indices (all data)	R1 = 0.0372, wR2 = 0.1021	R1 = 0.0286, wR2 = 0.0719	R1 = 0.1029, wR2 = 0.2627	R1 = 0.0718, wR2 = 0.1295	R1 = 0.0361, wR2 = 0.0931	R1 = 0.0565, wR2 = 0.1401
Extinction coefficient	0.034(2)	0.00020(5)	0.0036(7)	n/a	0.00118(17)	n/a
Largest diff. peak and hole (e.Å^{-3})	0.201 and −0.232	0.126 and −0.139	0.261 and −0.329	0.291 and −0.303	0.228 and −0.187	0.295 and −0.261

Table S2. CTE values comparison of PI-1 and PI-2 with the reported PIs.

Code	Dianhydride	Diamine	CTE(ppm K^{-1})	Reference
PI-1	8FDA	TFDB	14.5	-
PI-2	8FDA	TFODA	18.3	-
CPI-1	NTDA	APAB	3.0	[4]
CPI-2	NTDA	6ABO-4AB	12.9	[5]
CPI-3	PMDA	APAB	2.0	[6]
CPI-4	PMDA	ODA	4.25	[7]

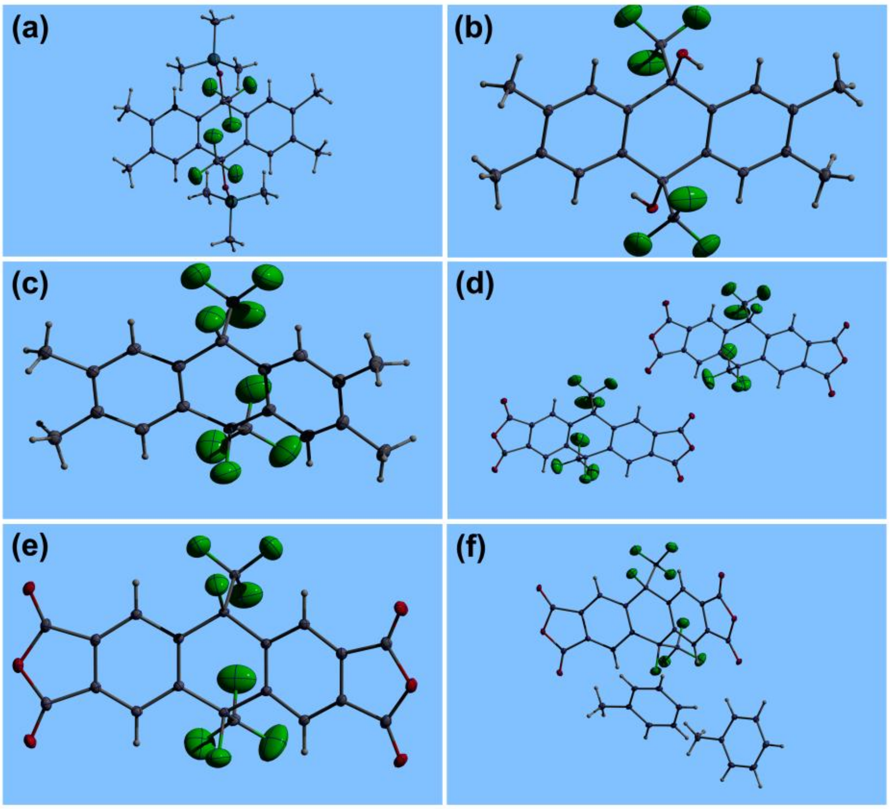

Figure S18. (a) The ORTEP molecular structures of compound **2**. (b) The ORTEP molecular structures of compound **3**. (c) The ORTEP molecular structures of compound **4**. (d) The ORTEP molecular structures of compound **6a**. (e) The ORTEP molecular structures of compound **6b**. (f) The ORTEP molecular structures of compound **6c**.

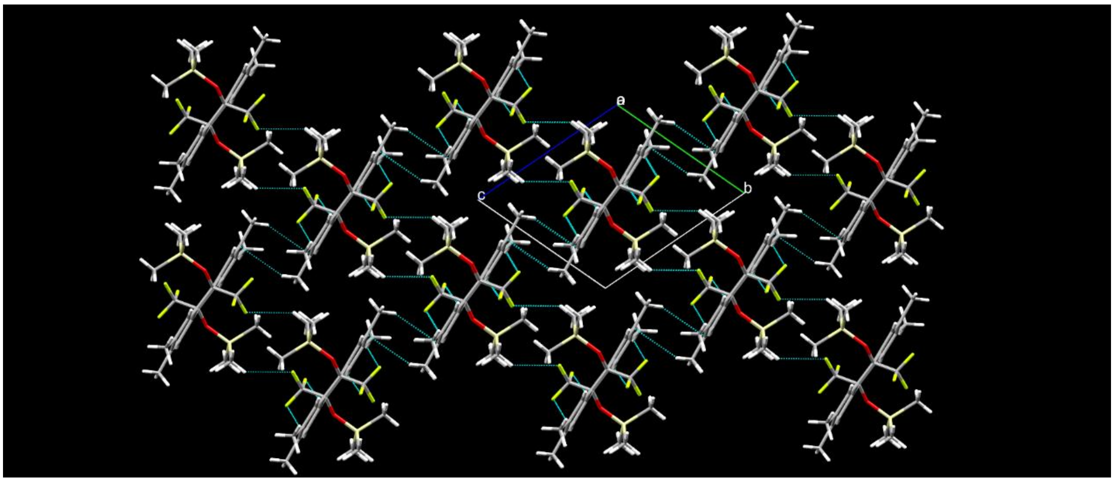

Figure S19. Part of the molecular packing in the nonsolvated 100(2) K Compound 2 crystals, with C–H…π interactions and C–F···H interactions shown as green dotted lines.

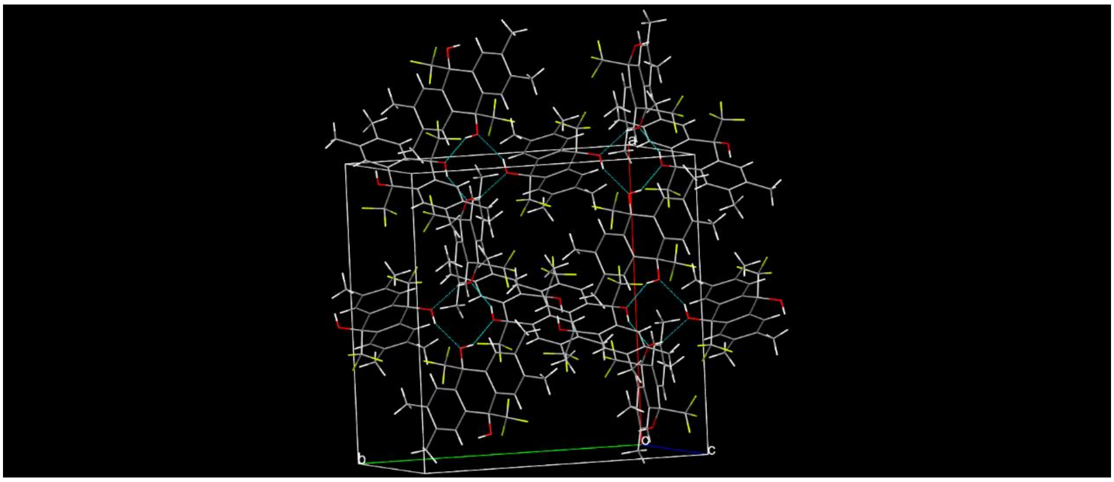

Figure S20. Part of the molecular packing in the nonsolvated 295(2) K Compound **3** crystals, with F…F and C–F…π interactions shown as green dotted lines.

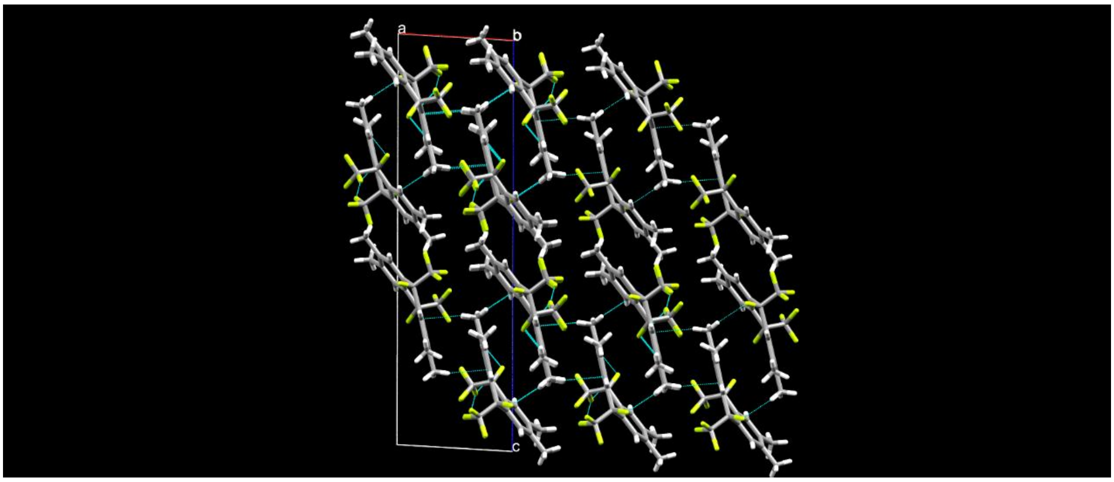

Figure S21. Part of the molecular packing in the nonsolvated 295(2) K Compound **4** crystals, with C–H…F interactions shown as green dotted lines.

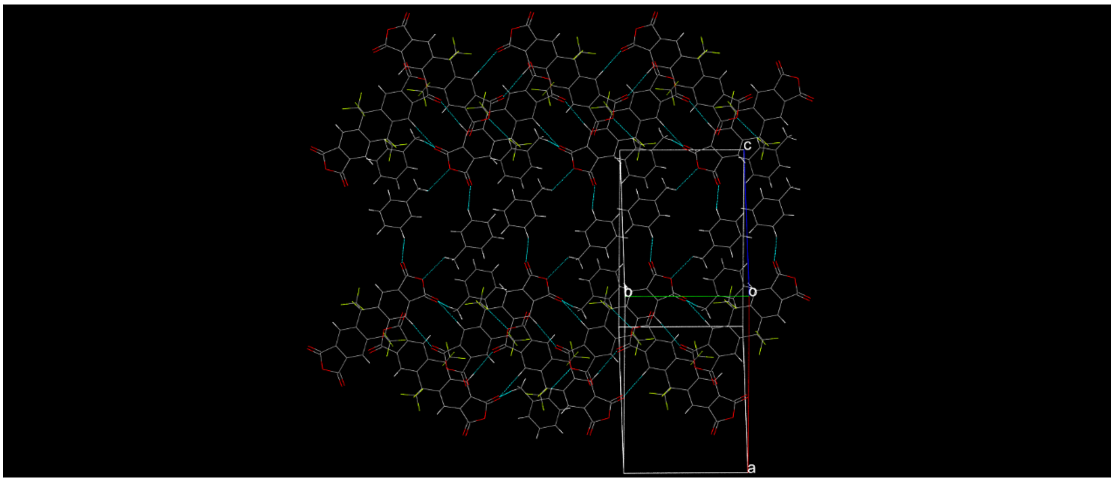

Figure S22. Part of the molecular packing in the nonsolvated 297(2) K Compound **6** crystals, with F…F, C–F…π and C–H…O interactions shown as green dotted lines.

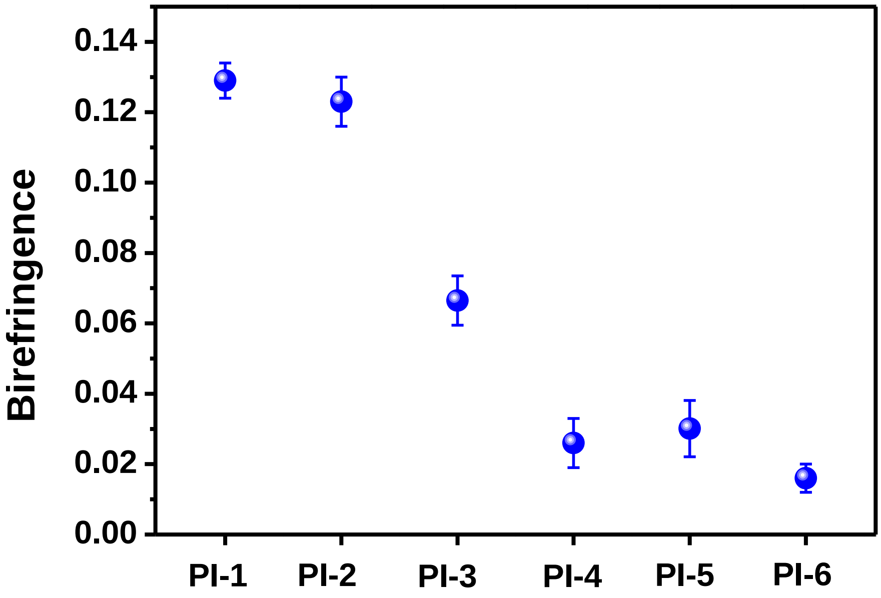

Figure S23. Birefringence data of polyimide films.

References

1. Wang, Z.; Guo, L.; Han, S.; Qi, H.; Cheng, Y.; Liu, F. Polyimides from an asymmetric hydroxyl-containing aliphatic-aromatic diamine synthesized via henry reaction. *J. Polym. Sci. Part A Polym. Chem.* **2017**, *55*, 3413–3423.
2. Kim, H.S.; Kim, Y.H.; Ahn, S.K.; Kwon, S.K. Synthesis and characterization of highly soluble and oxygen permeable new polyimides bearing a noncoplanar twisted biphenyl unit containing *tert*-butylphenyl or trimethylsilyl phenyl groups. *Macromolecules* **2003**, *36*, 2327–2332.
3. Li, J.; Zhang, H.; Liu, F.; Lai, J.; Qi, H.; You, X. A new series of fluorinated alicyclic-functionalized polyimides derivated from natural-(D)-camphor: Synthesis, structure–properties relationships and dynamic dielectric analyses. *Polymer* **2013**, *54*, 5673–5683.

4. Ebisawa, S.; Ishii, J.; Sato, M.; Vladimirov, L.; Hasegawa, M. Spontaneous molecular orientation of polyimides induced by thermal imidization (5). Effect of ordered structure formation in polyimide precursors on cte. *Eur. Polym.* **2010**, *46*, 283–297.
5. Hasegawa, M.; Hoshino, Y.; Katsura, N.; Ishii, J. Superheat-resistant polymers with low coefficients of thermal expansion. *Polymer* **2017**, *111*, 91–102.
6. Kotov, B.V.; Gordina, T.A.; Voishchev, V.S.; Kolninov, O.V.; Pravednikov, A.N. Aromatic polyimides as charge transfer complexes. *Polymer Science U.S.S.R.* **1977**, *19*, 711–716.
7. Bronnikow, S.V.; Sukhanova, T.E.; Goikhman, M.Y. Evolution of statistical ensemble of mierodomains on the surface of films of rigid-chain polymide during thermal imidezation. *Russion Journal of Applied Chemistry* **2003**, *76*, 967–971.
